# Mechanisms of electroacupuncture-induced neuroprotection in acute stroke rats: the role of astrocyte-mediated mitochondrial transfer

**DOI:** 10.1186/s12964-025-02287-9

**Published:** 2025-07-01

**Authors:** Yang Guo, Tiancong Fu, Yupei Cheng, Yuxuan Li, Runchen Zhang, Qingtao Ma, Guanran Wang, Wenhua Ning, Wen Fan, Juntao Yang, Mengxiong Zhao, Bohan Liu, Can Wang, Liang Gao, Zhifang Xu, Yi Guo, Xiaoyu Dai, Jiangwei Shi

**Affiliations:** 1 First Teaching Hospital of Tianjin University of Traditional Chinese Medicine, Tianjin, 300381 China; 2 National Clinical Research Center for Chinese Medicine Acupuncture and Moxibustion, Tianjin, 300381 China; 3 Tianjin University of Traditional Chinese Medicine, Tianjin, 301617 China; 4 Tianjin Academy of Traditional Chinese Medicine Affiliated Hospital, Tianjin, 300120 China; 5 The First Affiliated Hospital of Zhengzhou University, Zhengzhou, 450003 China; 6 Peking University Third Hospital, Beijing, 100191 China; 7 The TCM Hospital of Longquanyi, Chengdu, 610100 China

**Keywords:** Electroacupuncture, Stroke, Mitochondrial transfer, Astrocyte

## Abstract

**Background:**

Ischemic stroke significantly threatens human health, and current treatments remain limited, necessitating novel strategies. Mitochondrial transfer between neurons represents a crucial endogenous neuroprotective mechanism.

**Objective:**

This study investigated whether electroacupuncture enhances mitochondrial transfer from astrocytes to damaged neurons during acute cerebral ischemia, promoting neuroprotection.

**Methods:**

A middle cerebral artery occlusion (MCAO) model in Sprague-Dawley (SD) rats and an oxygen-glucose deprivation/reperfusion (OGD/R) model in vitro were employed. Neurobehavioral assessments, electron microscopy, multiplex immunofluorescence, tissue quantification, western blotting, qRT-PCR, transcriptomics, and proteomics were conducted to evaluate mitochondrial distribution, function, and intercellular transfer under electroacupuncture preconditioning and intervention.

**Results:**

Electroacupuncture significantly improved neurological outcomes and reduced brain tissue damage in MCAO rats. It facilitated mitochondrial transfer from astrocytes to neurons, increased functional mitochondria within neurons, and reduced neuronal apoptosis. These effects may involve regulation of the CD38-cADPR-Ca2 + signaling pathway and proteins associated with tunneling nanotubes (TNTs), such as F-actin, Miro1, TRAK1, and KIF5b.

**Conclusion:**

Electroacupuncture enhances mitochondrial transfer and function, exerting neuroprotective effects during acute ischemic stroke. This study highlights the potential of electroacupuncture as a therapeutic approach and identifies novel targets for brain protection strategies.

**Supplementary Information:**

The online version contains supplementary material available at 10.1186/s12964-025-02287-9.

## Introduction

Ischemic stroke is a leading global health concern, significantly impacting quality of life. According to the Global Burden of Disease (GBD) Study, ischemic stroke accounts for 62.4% of all stroke events worldwide [1]. China has the highest incidence, with approximately 2 million new cases annually, increasing at a rate of 9% per year [2, 3]. The primary causes include cerebral vascular stenosis, occlusion, or insufficient blood supply, leading to glucose and oxygen deprivation in affected brain tissue. This disrupts cellular homeostasis, causing pathological damage such as excitotoxicity, oxidative stress, neuroinflammation, and apoptosis [4, 5]. Maximizing neuronal survival post-stroke is, therefore, a key treatment goal.

Current treatment options are limited. The thrombolytic agent tPA, while effective, has a narrow therapeutic window (< 4.5 h) and carries bleeding risks, benefiting only ~ 5% of patients. Consequently, most patients receive only supportive care [6]. Moreover, promising experimental drugs, such as NXY-059, have failed in large-scale clinical trials [7]. These setbacks have led researchers to reexamine stroke mechanisms and explore innovative therapeutic approaches.

In 2011, Iadecola et al., brain’s endogenous capacity for self-preservation and emphasized the need to explore methods that activate these protective mechanisms [8]. One such mechanism, intercellular mitochondrial transfer, has emerged as an important endogenous neuroprotective process [9]. Under ischemic conditions, neurons release damaged mitochondria, which are taken up and recycled by astrocytes [10]. Conversely, astrocytes can transfer functional mitochondria back to neurons, aiding recovery [11]. This bidirectional exchange may represent a novel intercellular signaling mechanism. Among the identified pathways for mitochondrial transfer—tunneling nanotubes (TNTs), extracellular vesicles, and gap junctions—TNTs are the primary route [12–15]. TNTs, rich in filamentous actin (F-actin), form cytoplasmic bridges between cells and facilitate mitochondrial transport [16].

Acupuncture, recognized globally and applied in 183 countries and regions [17], has shown efficacy in stroke treatment [18]. Randomized controlled trials and preclinical studies suggest that acupuncture prevents and treats stroke by modulating cerebral blood flow, delaying ischemic cascade reactions, and regulating neurochemical release [19, 20]. Specifically, electroacupuncture preconditioning mimics ischemic preconditioning by increasing methionine enkephalin release and activating opioid receptors, reducing ischemia-reperfusion injury [21]. Additionally, post-stroke acupuncture regulates necrosis, apoptosis, and autophagy, exerting neuroprotective effects [22–24]. However, the relationship between electroacupuncture and endogenous protective mechanisms, particularly mitochondrial transfer, remains unclear.

We hypothesized that electroacupuncture promotes astrocytic release of functional mitochondria into damaged neurons, enhancing energy supply and exerting neuroprotective effects. Using Sprague-Dawley (SD) rats, we established in vivo middle cerebral artery occlusion (MCAO) and in vitro physical oxygen-glucose deprivation/reperfusion (OGD/R) models. By employing neurobehavioral assessments, electron microscopy, immunofluorescence staining, tissue quantification, western blotting, and quantitative real-time PCR (qRT-PCR), we investigated whether electroacupuncture regulates mitochondrial distribution, function, and intercellular transfer post-stroke. These findings aim to provide theoretical evidence for electroacupuncture as a therapeutic strategy for ischemic stroke and identify novel targets for brain ischemic injury.

## Methods

### Establishment of the MCAO rat model

Male SD rats were fasted for 12 h before surgery, but allowed free access to water. Anesthetize in an appropriate-sized induction chamber by using 5 L/min 3% isoflurane (RWD, China) flow rate in 30% O_2_/ 70% N_2_O mixture. Decrease flow rate to 1 L/min for maintenance. Rats were placed in the supine position, and the neck was shaved and disinfected. A 2 cm midline incision was made, and the common carotid artery (CCA), external carotid artery (ECA), and internal carotid artery (ICA) were isolated. The proximal ends of the CCA and ICA were ligated, and the ECA was clamped. A silicone-coated monofilament (RWD, China) was inserted into the CCA and advanced 18 mm to occlude the middle cerebral artery, the incision was sutured and disinfected, and the rats were returned to the animal facility after recovery. During the procedure, maintain the room temperature at around 25 °C and ensure the rats’ body temperature remains at an appropriate level of 37 ± 0.5 °C [25]. After model preparation, the laser speckle cerebral blood flow imaging system (RFLSI III) (RWD, China) was used to monitor and compare changes in blood flow within the bilateral middle cerebral arteries of rats post-modeling and before/after acupuncture intervention (Supplementary Fig. 5).

All procedures were approved by the Medical Ethics Committee of Tianjin University of Traditional Chinese Medicine and the First Teaching Hospital of Tianjin University of Traditional Chinese Medicine, following the National Institutes of Health’s Guide for the Care and Use of Laboratory Animals [Ethical Approval Number: TCM-LAEC201813].

### Primary neuron isolation and establishment of the OGD/R model

Brains were collected from 24-hour-old SD pups and placed in ice-cold Hank’s Balanced Salt Solution (HBSS) (Solarbio, China) for 5 min. The cerebral cortex was dissected, minced, and digested with 0.125% trypsin at 37 °C for 20 min. Digestion was halted with ice-cold fetal bovine serum (FBS) (Gibco, USA), and cells were washed 2–3 times with Phosphate buffered saline (PBS) (Solarbio, China). The cell suspension was gently triturated, and the supernatant was collected while discarding undigested tissue. Cells were seeded into culture flasks, and after 4 h, the medium was replaced with DMEM/F12K (Hyclone, USA) supplemented with 10% FBS and 1% penicillin-streptomycin (HyClone, USA). On the second day, 10 µmol/L 5-fluorouracil (5-FU) (MedChemExpress, USA) was added, and half of the medium was replaced every 3 days.

For the OGD/R model, cultured neurons were washed three times with glucose-free DMEM, which was then replaced. The cells were incubated in a sealed chamber perfused with 95% N₂ and 5% CO₂ at 1 L/min for 15 min. Once the oxygen level dropped below 0.1%, the chamber was sealed and incubated at 37 °C for 60 min. After hypoxia, the chamber was vented, and the medium was replaced with complete DMEM o restore normal oxygen and glucose levels.

### Electroacupuncture pretreatment and intervention

Electroacupuncture was administered using disperse-dense waves at a frequency of 2/10 Hz, with a 2 mA current for 20 min. Sterile disposable acupuncture needles (0.25 mm × 25 mm and 0.25 mm × 13 mm) (Yuwell, China) were used at the following acupoints: Baihui (GV20), Neiguan (PC6), and Sanyinjiao (SP6), located per the “Atlas of Common Acupoint Locations in Experimental Animals” and the national standard “Names and Locations of Acupoints” (GB/T12346-2006). Specifically, Baihui is located at the midline of the parietal bone; Neiguan is located between the radius and ulna on the medial side of the forelimb, approximately 3 mm above the wrist joint; and Sanyinjiao is located approximately 5 mm above the medial malleolus of the hind limb.

Rats were randomly assigned to groups using a computer-generated randomization protocol. For pretreatment, groups received one, three, or five electroacupuncture sessions, with ischemic modeling performed 24 h after the final session. For intervention, treatments were administered 6, 12, 18, and 24 h post-modeling.

### Neurological behavioral assessment

#### Modified neurological severity score (mNSS)

Motor, sensory, reflex, and balance functions were scored (range: 0–18), with higher scores indicating more severe neurological deficits. Assessments were performed at specific time points by blind observers.

#### Garcia score

Six parameters (spontaneous activity, limb movement symmetry, forepaw extension, climbing ability, proprioception, vibrissa response) were scored (range: 0.0–18), with higher scores indicating better neurological function. The observers were blinded to group assignments.

### Triphenyltetrazolium chloride (TTC) staining for brain infarct volume

Following anesthesia, the rats were rapidly decapitated, and the intact brain tissue was removed and placed on ice. The brains were frozen at − 20 °C for 20 min. Using a brain matrix, the tissue was coronally slice into five sections, each ~ 2 mm thick. The slices were stained with 2% 2,3,5-triphenyltetrazolium chloride (TTC) (Solarbio, China) at 37 °C for 30 min, and fixed in 4% paraformaldehyde (Solarbio, China) for 24 h. Infarct volumes were quantified using ImageJ 1.51 software to measure the area of contralateral (healthy) and the ipsilateral (affected) sides. The infarcted volume was calculated as:

*A* = Area of contralateral brain tissue − Area of normal brain tissue on affected side.

The infarct volume was calculated by summing the infarct areas of all slices and multiplying by slice thickness (t):

$$\:{V}_{infarct}\:=\left(A1+A2+A3+A4+A5\right)\:\times\:\:t$$

The volume of contralateral brain tissue was calculated as:

$$\:{V}_{contralateral}\:=\left(Area\:of\:contralateral\:brain\:tissue\right)\:\times\:\:t$$

The infarct volume on the affected side was calculated by summing the infarct areas of all slices and multiplying them by the slice thickness as follows:

$$\:Infarct\:Volume\:Percentage=\left(\frac{{V}_{infarct}}{{V}_{contralateral}}\right)\:\times\:\:100\:$$

### Apoptosis rate of neural cells (TUNEL staining)

After were anesthetized, and the chest cavity was opened to expose the heart. A perfusion needle was inserted into the ascending aorta, and the right atrial appendage was cut. The rats were perfused sequentially with 200 ml of 0.9% saline, followed by 200 ml of 4% paraformaldehyde (Solarbio, China) over approximately 25 min, and brains extracted. Coronal sections, spanning 2 mm anterior and posterior to the optic chiasm, were prepared, and the middle portion of the tissue was fixed overnight in 4% paraformaldehyde.

Fixed brain tissues were dehydrated, cleared, and embedded in paraffin. Serial Sect. (5 μm thick) were cut, and every fifth section was collected, focusing on the ischemic hippocampal region. Sections were deparaffinized using xylene (Sinopharm Chemical Reagent Co., Ltd, Chian) and rehydrated through a graded ethanol series. After membrane permeabilization, TUNEL working solution (a 1:9 mixture of TdT and dUTP) (Roche. Switzerland) was applied dropwise, and the sections were incubated at 37 °C in the dark for 2 h. DAPI staining solution (Biosscn, Chian) was subsequently added, and the sections were incubated at room temperature in the dark for 10 min before mounting.

Images were captured at 200× magnification using a fluorescence microscope (Olympus, Japan). DAPI-stained nuclei appeared blue, while TUNEL-positive nuclei appeared green. Image analysis was performed using ImageJ 1.51 software to determine the apoptosis rate, calculated as the percentage of TUNEL-positive cells relative to the total number of nuclei:

$$\:Apoptosis\:Rate=\left(\frac{TUNEL-positive\:nuclei}{Total\:nuclei}\right)\:\times\:\:100$$

### Observation of neural cell ultrastructure by transmission electron microscopy (TEM)

After anesthetizing and decapitating the rats, ischemic penumbral tissue was quickly extracted and cut into pieces approximately 1 mm^3^. Samples were fixed with 2.5% glutaraldehyde at 4 °C for 24 h. Following fixation, the samples were rinsed three times with PBS buffer and transferred to 1% osmium tetroxide for fixation at 4 °C for 1 h. The samples were then washed with double-distilled water and dehydrated at 4 °C using a graded ethanol series (50, 70, 80, and 90%) (Sinopharm Chemical Reagent Co., Ltd, Chian) for 5 min each. Further dehydration was carried out at room temperature using 90%, 95%, and 100% acetone (Sinopharm Chemical Reagent Co., Ltd, Chian) for 5 min each, followed by clearing in 100% acetone for 20 min.

The dehydrated samples were embedded in epoxy resin and shaken at 40 °C for 6 h to ensure thorough penetration. Polymerization was performed by baking the samples in ovens at 50 °C, 60 °C, and 72 °C, each for 24 h, to harden the embedding medium. The hardened blocks were trimmed, and semi-thin Sect. (0.5–1 μm thick) were prepared and stained with toluidine blue for tissue localization under a light microscope (Leica, Germany). Ultrathin sections (~ 60 nm thick) were cut and mounted onto copper grids.

Sections were stained with uranyl acetate (Solarbio, China) for 20 min, rinsed with water, and dried. The cells were then counterstained with lead citrate in the dark for 15 min, followed by rinsing and dying. Finally, the sections were examined under a transmission electron microscope (TEM) (hitachi, Japan). For each sample, three fields were randomly selected for photography and ultrastructural analysis.

### Multiple immunofluorescence staining

Paraffin-embedded sections were deparaffinized and hydrated by immersing them in fresh xylene three times for 10 min each. The sections were then treated with graded ethanol solutions (100%, 95%, and 70%) for 5 min each and washed three times with sterilized water for 1 min each. After fixation in 10% neutral formalin for 10 min, antigen retrieval was performed using microwave heating. Sections were placed in an antigen retrieval solution, heated at high power, and maintained at low power for 15 min before cooling to room temperature.

A blocking agent was applied for 10 min at room temperature to reduce nonspecific binding. Diluted primary antibodies were then added, and the sections were incubated at 37 °C for 1 h, followed by washing with 1×TBST buffer (Solarbio, China). Secondary antibodies were applied, and the sections were incubated at room temperature for 10 min before another wash. A fluorophore working solution was added to enhance the fluorescence signal, and the sections were incubated for 10 min at room temperature.

After washing, microwave antigen retrieval was repeated, and the sections were cooled and washed again. The blocking, primary antibody, secondary antibody (Abcam, Britain), and staining steps were repeated for multiple stains. Finally, DAPI working solution was added, and the sections were incubated at room temperature for 5 min before washing. The sections were mounted using an anti-fluorescence quenching mounting medium, covered with a coverslip, and sealed. Fluorescence microscopy was used to observe the sections, and statistical analyses were performed based on the acquired images.

### Panoramic tissue quantitative analysis

Quantitative analysis of the panoramic tissue was conducted to statistically evaluate the results of multiple immunofluorescence staining. Image analysis software was used to identify cell nuclei in the DAPI channel fluorescence channel (430 nm). The cytoplasmic area of each cell was delineated using corresponding fluorescence channels (520 nm for NeuN and 700 nm for GFAP). The area and average fluorescence intensity of each cell were calculated.

Scatter plots were generated with the cell area on the x-axis and the average fluorescence intensity on the y-axis. Using the software’s retrospective tracking function, combined with cellular morphology and fluorescence characteristics, thresholds were applied to distinguish neurons (NeuN-positive cells) and astrocytes (GFAP-positive cells) from the total cell population.

Among the identified neurons and astrocytes, cells positive for mitochondria (620 nm channel) were further analyzed. The fluorescence intensity of mitochondria within the cytoplasm of these cells was calculated to reflect the mitochondria abundance. Cells expressing mitochondria were identified using the retrospective tracking function and mitochondrial fluorescence images.

Statistical analyses were performed to compare the proportion of mitochondria near neurons and astrocytes across the sham-operated, model, and acupuncture groups providing insights into mitochondrial distribution and activity.

### Mitochondrial extraction and labeling

High-purity, functionally intact mitochondria were extracted from the rat brain tissue using a mitochondrial isolation kit (Beyotime, China), following the manufacturer’s instructions. Solutions prepared by dissolving components at room temperature and mixing them on ice. For initial preparation, 1.5 ml of PMSF solvent (Beyotime, China) was added to PMSF crystals to create a 100 mM stock solution, which was stored at − 20 °C. During the extraction process, PMSF was added to the mitochondrial samples along with mitochondrial lysis buffer to achieve a final PMSF concentration of 1 mM.

Fresh rat brain tissue collected within one h post-mortem, was processed on ice. Approximately 50–100 mg of tissue was weighed, placed in a 1.5 ml centrifuge tube, and washed once with PBS. Pre-cooled mitochondrial isolation reagent A (10x tissue volume) (Beyotime, China) was added, and the tissue was homogenized on ice approximately 10 times. The homogenate was centrifuged at 600 × g at 4 °C for 5 min. The supernatant was discarded, and the pellet was collected and centrifuged again at 11,000 g for 10 min at 4 °C, yielding the mitochondrial fraction.

For fluorescence labeling and functional studies, the mitochondrial pellet was resuspended in 40 µl of mitochondrial storage solution. Mitochondria were labeled with MitoTracker Red by diluting a 1 mM stock solution in PBS to the appropriate working concentration. The mitochondrial suspension was incubated with the MitoTracker Red working solution at 37 °C with 5% CO₂ for 30 min. After staining, labeled mitochondria were immediately injected into specific regions of the rat brain using a stereotaxic apparatus for subsequent immunofluorescence experiments and analyses.

### Stereotactic brain injection

Astrocytes or fluorescently labeled mitochondria were stereotactically injected into specific brain regions of rats. Rats were anesthetized using an gas anesthesia machine (RWD, China) and reached a deep anesthesia after approximately 10 min. The head was secured in a stereotaxic apparatus to ensure secure accurate fixation and symmetrical alignment, maintaining the precision of the injection site.

The surgical area was prepared and disinfected, and a midline incision was made to expose the skull. Connective tissue at the anterior and posterior fontanelles was cleared, and the skull surface was rinsed with saline. To locate the injection site, the syringe was positioned at the anterior fontanelle, touching the skull surface to establish the anterior fontanelle as the zero point (AP: 0.00 mm, ML: 0.00 mm, DV: 0.00 mm). The head was adjusted to align the sagittal suture and level the anterior-posterior and left-right orientations.

According to the stereotaxic atlas, the injection point was identified at AP: − 1.00 mm, ML: 1.50 mm, DV: 0.00 mm. A bone-hole (~ 1 mm²) was drilled at this location. A microsyringe was filled with 5 µL of freshly fluorescently labeled mitochondrial solution and inserted into the brain area at AP: − 1.00 mm, ML: 1.50 mm, DV: − 2.00 mm. The solution was injected at 1 µL per minute, with 0.1 µL injected initially, followed by a 1-min pause, and subsequent 0.1 µL increments until completion. After injection, the needle was left in place for 5 min to prevent backflow before being slowly withdrawn.

The incision was sutured, and the wound was disinfected with iodophor. The rats were placed in a warm box to recover and returned to their cages upon regaining full consciousness.

### Cell viability detection by CCK-8 assay

Cells were adjusted to a density of 1 × 10⁴ cells per well and evenly seeded into 96-well plates (Corning, USA). The cells were incubated overnight at 37 °C with 5% CO₂ to facilitate cell attachment. Following hypoxic treatment under OGD/R model conditions, the cells were reoxygenated for 24 h. Then, 10 µL of CCK-8 reagent (prepared at a volume ratio of 1:9 with culture medium) (Sigma-Aldrich, Germany) was added to each well and the plates were incubated at 37 °C for 2 h. After incubation, 100 µL of supernatant from each well was transferred to a new 96-well plate. Absorbance was measured at 450 nm using a microplate reader to evaluate cell viability.

### Annexin V-FITC/PI double staining for apoptosis rate detection

Primary neurons were seeded into 6-well plates (Corning, USA) at a density of 5 × 10⁵ cells per well and incubated overnight at 37 °C with 5% CO₂. After OGD conditions, the cells were reoxygenated for 24 h. The culture supernatant was collected, and the cells were washed once with PBS. Cells were digested with 0.25% trypsin (HyClone, USA) at room temperature until detachment began, and digestion was halted with culture medium. The cell suspension was gently pipetted, transferred to centrifuge tubes, and centrifuged at 1,000 rpm for 10 min. The supernatant was discarded, and the cells were resuspended in PBS and counted. Approximately 50,000–100,000 cells were transferred to a fresh tube and resuspended in 195 µL of Annexin V-FITC binding buffer (Beyotime, China). Next, 5 µL each of Annexin V-FITC and propidium iodide (PI) (Solarbio, China) staining solution were added. The cells were gently mixed and incubated at room temperature in the dark for 10–20 min. After staining, 200 µL of binding buffer was added, and the samples were immediately analyzed by flow cytometry to determine apoptosis rate.

### Lactate dehydrogenase (LDH) release assay

Cells were seeded onto plates based on experimental requirements and treated as specified. After the designated incubation period, supernatants were collected from each well. Following the protocol of a commercial LDH assay kit (Sangonbio, China), colorimetric reagents were added, and the mixture was incubated in the dark at room temperature for 5 min. Absorbance was measured at 450 nm. Untreated cells served as negative controls, while completely lysed cells were used as positive controls. The LDH release rate, indicative of cytotoxicity, was calculated by comparing the absorbance of treated samples to the controls.

### Proteomics analysis

Proteomic analysis was performed using isobaric tags for relative and absolute quantitation (iTRAQ) (AB SCIEX, USA) labeling combined with high-performance liquid chromatography-tandem mass spectrometry (HPLC-MS/MS) (AB SCIEX, USA). Proteins were extracted from tissue samples using the SDT lysis method, and concentrations were determined and validated via SDS-PAGE. Equal amounts of protein were digested with trypsin using the filter-aided sample preparation (FASP) method.

Peptides were purified using a C18 desalting column and labeled with iTRAQ reagents. Labeled peptides were mixed and fractionated using strong cation-exchange (SCX) chromatography. Fractions were separated using nano-liquid chromatography and analyzed with a Q-Exactive mass spectrometer (Thermo Scientific, USA).

Mass spectrometry data were processed and quantified using Mascot 2.2 and Proteome Discoverer 1.4 software (Thermo Scientific, USA). Bioinformatic analyses included Gene Ontology (GO) functional annotation, Kyoto Encyclopedia of Genes and Genomes (KEGG) pathway analysis, enrichment analysis, clustering analysis, and construction of protein-protein interaction networks.

### Transcriptomic analysis

Transcriptomic analysis was performed via RNA sequencing (RNA-seq). Total RNA was extracted, and quality was assessed using RNA-specific agarose gel electrophoresis and an Agilent 2100 Bioanalyzer. mRNA was isolated from total RNA by exploiting its poly(A) tail specificity and fragmented into 200–300 base pairs (bp) (Bio-RAD, USA).

Using fragmented mRNA as a template, cDNA was synthesized to construct a cDNA library. Target fragments were enriched by PCR, and the library size was adjusted to 300–400 bp. High-throughput sequencing was conducted using the Illumina sequencing platform.

Raw sequencing data underwent quality control to remove low-quality reads and adapter sequences, resulting in high-quality clean data. Clean reads were aligned to a reference genome, and differentially expressed genes (DEGs) were identified using|log₂FoldChange| > 1 and *P* < 0.05 as criteria. GO and KEGG functional annotation and enrichment analyses were performed on DEGs to identify key biological processes, cellular components, molecular functions, and signaling pathways.

### Western blot analysis

Total protein was extracted from tissues or cells using RIPA lysis buffer (Solarbio, China), and concentrations were determined using the bicinchoninic acid (BCA) method (Beyotime, China). Equal amounts of protein were separated by sodium dodecyl sulfate-polyacrylamide gel electrophoresis (SDS-PAGE) (Solarbio, China) and transferred onto polyvinylidene difluoride (PVDF) membranes (Millipore, USA).

Membranes were blocked with 5% non-fat milk blocking solution for 1 h. Primary antibodies (Bioss, China) were applied, and membranes were incubated overnight at 4 °C. After washing with TBST buffer (Solarbio, China), secondary antibodies (Bioss, China) were applied, and the membrane was incubated for 2 h at room temperature.

Proteins were detected using HRP chemiluminescence reagents (Bioss, China), with exposure times adjusted as needed. Grayscale analysis was conducted to quantify the expression levels of target proteins relative to the internal control.

### qRT-PCR detection

Total RNA was extracted from the ischemic side of the brain tissue using TRIzol reagent (TIANGEN, China) following standard procedures. RNA concentration and purity were measured with a NanoDrop 2000 spectrophotometer (Thermo, USA), and integrity was assessed using 1.5% agarose gel electrophoresis (Bio-RAD, USA). cDNA synthesis was performed using Oligo(dT)₁₈ primers and RevertAid M-MuLV reverse transcriptase (Thermo, USA). The analyzed genes included: F-actin (F: CAGAGGATGTGGATGGAGGCTTG, R: TTTTGGGTTGAAACTGGTGGCTT), Miro1 (F: TGGAAGAAACCTAATGAGGCAGAAG, R: TACCAGGCATACAACATCACAAACG), TRAK1 (F: CTTCTATGTGCCGAAAGAGTTGG, R: TTCCTCTCAGTTAGGGTCTTGTTCT), KIF5a (F: CGGCACCTGGAAGAGTCCTATG, R: CCTGCGTATCTGGCTCCTTGTC), KIF5b (F: CAAGTATGTCGCCAAGTTCCAGG, R: GGGTCTTTCCAGATGATGTTTGTCC), KIF5c (F: GATGAGGGCAAAGCAAACCGAC, R: ACTCCCAGCCAAATCAACCAAATAC), and Internal control gene GAPDH (F: TTCAGCTCTGGGATGACCTT, R: TGCCACTCAGAAGACTGTGG). The synthesized cDNA was diluted and used as a template for qPCR amplification with a SYBR Green detection system. The qPCR program consisted of an initial denaturation at 95 °C for 30 s, followed by 40 cycles of 95 °C for 5 s (denaturation), 60 °C for 30 s (annealing), and 72 °C for 20 s (extension). A melting curve analysis was conducted to confirm amplification specificity. Ct values were analyzed using StepOne software, and relative gene expression levels were calculated using the 2^-ΔΔCt method, with GAPDH serving as the internal control.

### Measurement of intracellular ca²⁺ concentration

Prepared astrocytes were incubated with a Fluo-3 AM/DMSO solution (Solarbio, China) containing 16.5 mg of Pluronic F127 to facilitate dye entry into cells and prevent aggregation in HBSS. The solution was diluted with HBSS to a final Fluo-3 AM concentration of 4–5 µM to prepare the working solution. The working solution was added to the cells and incubated at 37 °C for 20 min. Subsequently, HBSS containing 1% fetal bovine serum (5x the volume of the working solution) was added, and incubation was continued for another 40 min. The cells were washed three times with HEPES-buffered saline (Solarbio, China) solution and resuspended in the same buffer at a concentration of 1 × 10⁵ cells/mL. After equilibration at 37 °C for 10 min, intracellular Ca²⁺ levels were measured using fluorescence detection at an excitation wavelength of 506 nm and an emission wavelength of 526 nm.

### Statistical analysis

All statistical analyses were performed using SPSS software (version 26.0). Data normality was assessed using normality tests. Normally distributed data were expressed as mean ± standard deviation (mean ± SD) and analyzed using one-way analysis of variance (ANOVA). For homogenous variances, LSD post-hoc tests were applied, while Dunnett’s T3 test was used for heterogeneous variances. Non-normally distributed data were expressed as median (25th percentile, 75th percentile) *M*(*P*_25_,*P*_75_) and analyzed with non-parametric tests, including the Kruskal-Wallis H test. Behavioral data were evaluated using the Kruskal-Wallis test for group differences and the Mann-Whitney U test for pairwise comparisons. Statistical significance was set at *P* < 0.05.

## Results

### Temporal patterns of neuronal protection by electroacupuncture (EA) pretreatment and intervention in MCAO rats

Establishing a reliable and stable acupuncture platform is foundational for investigating the neuroprotective mechanisms of EA. This study explored time- and dose-dependent relationships of EA preconditioning and intervention to clarify their neuroprotective effects and guide future research.

**Fig. 1:**
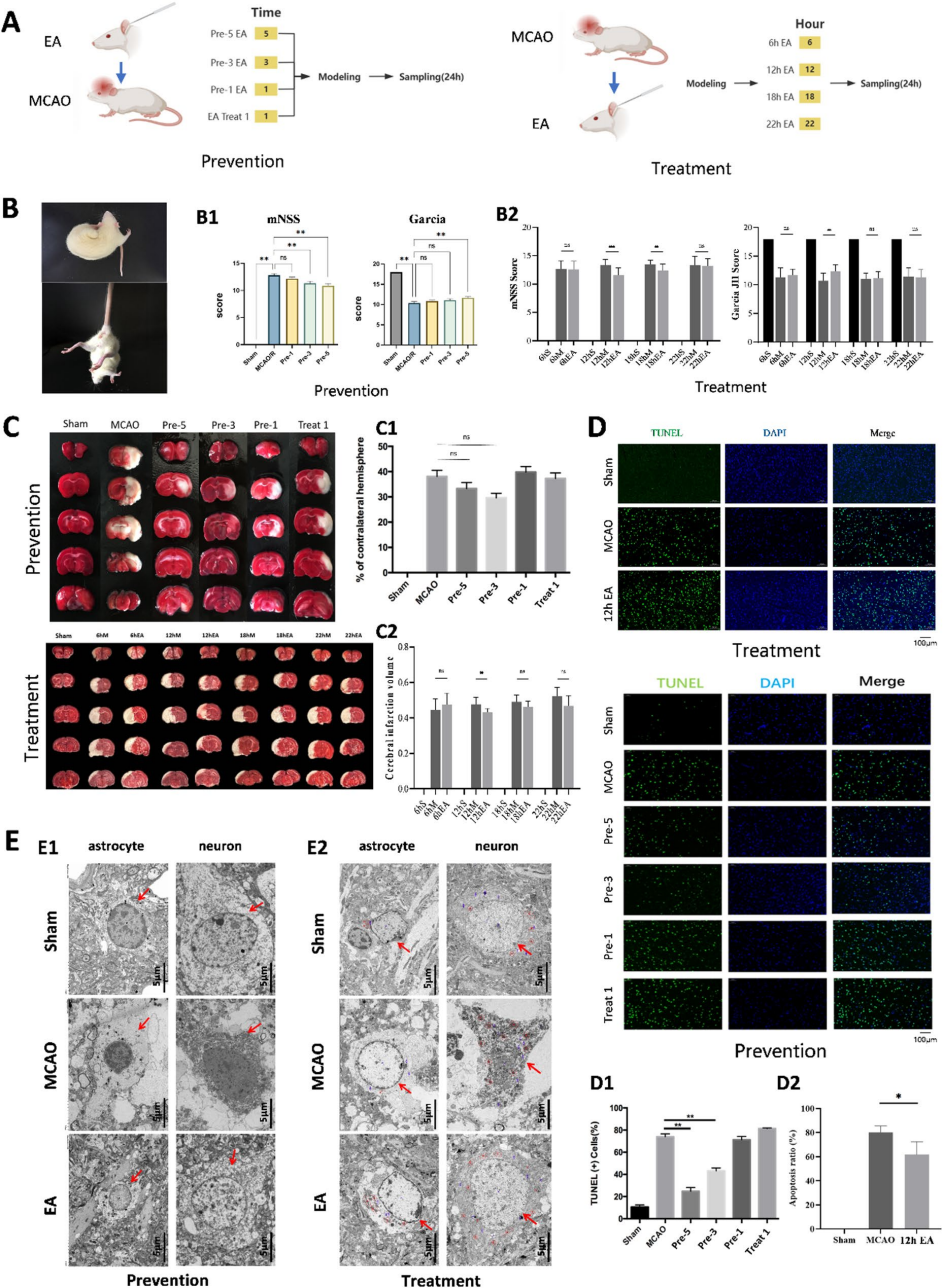
Study on the time-effect relationship of EA pretreatment and EA intervention for neuronal protection in MCAO model rats. **(A)** Timeline of EA pretreatment and EA intervention. **(B)** B1: The effects of EA intervention at different time points on the mNSS and Garcia JH scores in MCAO model rats (*n* = 12 per group); B2: The effects of different numbers of EA pretreatments on the mNSS and Garcia JH score in MCAO model rats (*n* = 10 per group). **(C)** Representative TTC-stained images of MCAO rats. C1: The effects of different numbers of electroacupuncture pretreatments on the infarct volume in MCAO model rats (*n* = 7 per group) C2: The effects of electroacupuncture intervention at different time points on the infarct volume in MCAO model rats (*n* = 10 per group). S, sham operation group; M, model group; EA, electroacupuncture group (ns: *P* > 0.05; *: *P* < 0.05; **: *P* < 0.01; ****: *P* < 0.001). **(D)** The effects of electroacupuncture intervention and electroacupuncture pretreatment on the number of TUNEL-positive cells in the ischemic penumbra cortex of MCAO rats. Green: TUNEL; Blue: DAPI; merge: TUNEL + DAPI. D1: The effects of different numbers of electroacupuncture pretreatments on the apoptosis rate of neurons in the infarcted brain tissue of MCAO model rats (*n* = 5 per group). D2: The effects of electroacupuncture intervention 12 h after modeling on the apoptosis rate of neurons in the infarcted brain tissue of MCAO model rats (*n* = 4 per group). (One-way ANOVA was used for comparison among multiple groups, and Tukey’s post hoc test was used for pairwise comparison. ns: *P* > 0.05; *: *P* < 0.05; **). **(E)** E1: Electron micrographs showing the effects of EA intervention on the morphology and structure of neurons and astrocytes in the ischemic penumbra of the cerebral cortex in MCAO model rats (*n* = 4 per group). E2: Electron micrographs showing the effects of EA pretreatment on the morphology and structure of neurons and astrocytes in the ischemic penumbra of the cerebral cortex in MCAO model rats (*n* = 4 per group). **Note**: EA Acupoints: Baihui (GV20), bilateral Neiguan (PC6), and Sanyinjiao (SP6). EA Parameters: Sparse-dense waves: 2/10 Hz, Intensity: 1 mA, Duration: 20 min

In the EA pretreatment experiments, rats were random divided into groups receiving one, three, or five sessions of pretreatment (*n* = 12 per group). Neurological and behavioral assessments revealed significant improvements in mNSS and Garcia scores in all EA groups compared to the MCAO group, with the 5-times group showing the greatest improvement (*P* < 0.01) (Fig. 1, A1–B1) TTC staining revealed reduced cerebral infarct volumes in all pretreatment groups, with the three-times pretreatment group showing the most noticeable reduction, although no statistical differences were observed (*n* = 7 per group) (Fig. 1, C1). TUNEL immunofluorescence significantly fewer TUNEL-positive cells in the ischemic penumbra of the three- and five-times pretreatment groups compared to the MCAO group (*n* = 5 per group, *P* < 0.05) (Fig. 1, D1). Electron microscopy showed that the MCAO group exhibited severe neuronal and astrocytic damage, including cell membrane rupture, mitochondrial swelling, chromatin loss, and organelle disruption. In contrast, EA pretreatment groups demonstrated milder damage, with intact membranes, slight edema, and fewer autophagolysosomes (*n* = 4 per group) (Fig. 1, E1).

In the EA intervention experiments, treatments were applied at 6, 12, 18, or 22 h post-MCAO (*n* = 10 per group). Neuronal assessments revealed that the 12-h intervention group exhibited the most significant improvements in mNSS and Garcia scores (*P* < 0.01), indicating that this time point provided optimal neuroprotection (Fig. 1, A2-B2). TTC staining confirmed a significant reduction in cerebral infarct volumes in the 12-h group (*n* = 10 per group, *P* < 0.01) (Fig. 1, C2) and TUNEL staining revealed fewer apoptotic cells in the ischemic penumbra (*n* = 4 per group, *P* < 0.05) (Fig. 1, D2). Electron microscopy of the 12-h group showed mild-to-moderate damage to neurons and astrocytes including intact cell membranes, moderate organelle swelling, and fewer autophagolysosomes (*n* = 4 per group) (Fig. 1, E2). Both EA pretreatment and intervention were found to protect neuronal synapses and the blood-brain barrier integrity in the ischemic penumbra of the cerebral cortex in MCAO rats (Supplementary Fig. 1).

Based on these findings, five sessions of EA pretreatment and 12-h post-MCAO intervention were chosen for subsequent experiments.

### Regulation of mitochondrial distribution, function, and morphology by EA intervention in MCAO rats

**Fig. 2:**
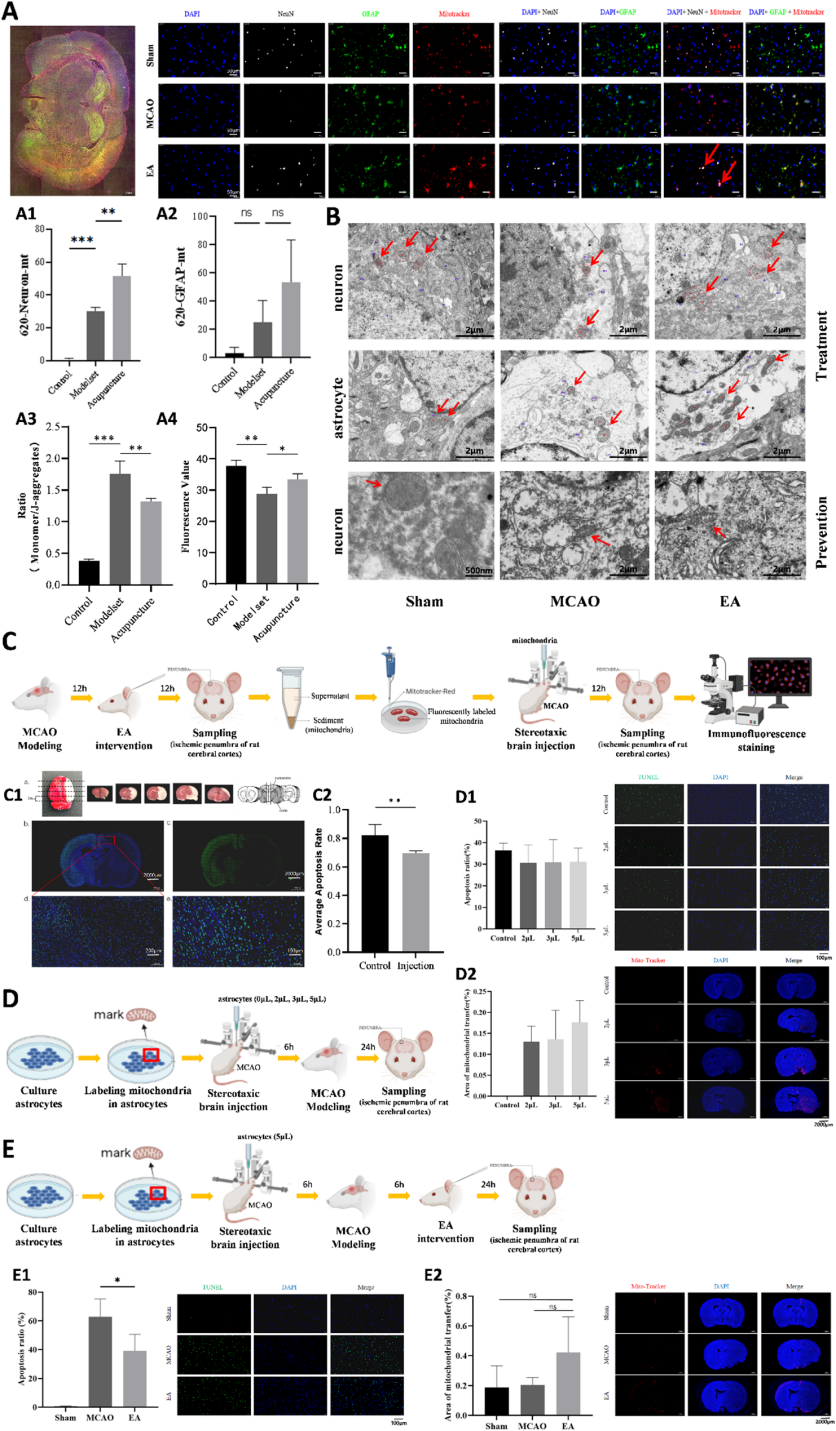
Effects of EA on the distribution, functional activity and morphology of mitochondria in the brain of MCAO rats. **(A)** Multiple immunofluorescence staining, panoramic tissue quantitative analysis and the effects of EA intervention on mitochondrial functional activity. A1: Effects of EA intervention on mitochondrial aggregation near neurons in the ischemic penumbra of the cerebral cortex of MCAO rats (*n* = 4 per group). A2: Effects of EA intervention on mitochondrial aggregation near astrocytes in the ischemic penumbra of the cerebral cortex of MCAO rats (*n* = 4 per group). A3: Effects of EA intervention on mitochondrial membrane potential in the ischemic penumbra of the cerebral cortex of MCAO rats (*n* = 4 per group). A4: Effects of EA intervention on mitochondrial activity of neurons in the ischemic penumbra of the cerebral cortex of MCAO rats (*n* = 4 per group). **(B)** Effects of EA intervention and EA pretreatment on the morphological structure of mitochondria near neurons and astrocytes in the ischemic penumbra of the cerebral cortex of MCAO rats (*n* = 4 per group). **(C)** Study on the process of EA regulating mitochondrial transcellular transfer based on stereotaxic brain injection. C1: Mitochondrial aggregation in the brain of MCAO model rats (Figure a shows the TTC staining results of the whole brain and brain slices; Figure b shows the results of brain tissue immunofluorescence staining, blue is DAPI, green is Tunel cell apoptosis, the scale bar is 2000 μm, and Figure d shows the results of its enlarged scale bar of 200 μm; Figure e shows the results of brain tissue immunofluorescence staining, blue is DAPI, green is NeuN (neuron), the scale bar is 200 μm, and Figure c shows the results of its reduced scale bar of 2000 μm. As can be seen from the figure, the number of neurons on the affected side is significantly less than that on the healthy side). C2: The effect of active mitochondria after acupuncture injection on the apoptosis rate of nerve cells in MCAO model rats (**: *P* < 0.01) (*n* = 5 per group). **(D)** The effect of different doses of astrocytes injected into the brain on the distribution of mitochondria in the brain of MCAO model rats. D1: The effect of different doses of astrocytes injected on the apoptosis rate of neurons in MCAO rats (*n* = 5 per group). D2: The effect of different doses of astrocytes injected on the distribution area of ​​mitochondria in the brain (*n* = 5 per group). **(E)** The effect of EA on mitochondrial distribution in the brains of MCAO model rats injected with 5 μm-sized astrocytes. E1: The effect of EA on neuronal apoptosis in MCAO rats injected with astrocytes (*n* = 5 per group). E2: The effect of EA on the area of mitochondrial distribution in the brains of MCAO rats injected with astrocytes (*n* = 5 per group)

To evaluate mitochondrial aggregation, function, and morphology in the ischemic penumbra, multiple immunofluorescence staining, high-precision scanning, and electron microscopy were employed. Tissue cytometry analysis revealed a significant increase in mitochondrial content near neurons in the EA-treated group compared to the model group (*P* < 0.01), while mitochondrial content near astrocytes showed a non-significant increasing trend (*n* = 4 per group) (Fig. 2, A1–A2). EA intervention also improved mitochondrial function, as evidenced by higher mitochondrial membrane potentials (*P* < 0.01), and ATP levels (*P* < 0.05) in the ischemic penumbra (*n* = 4 per group) (Fig. 2, A3–A4). Furthermore, EA treatment reduced mitochondrial damage, as reflected by decreased reactive oxygen species (ROS) fluorescence intensity, calcium ion concentrations, and mtDNA damage, thereby preserving mitochondrial integrity and activity (Supplementary Fig. 2).

Electron microscopy revealed that mitochondria near neurons and astrocytes in the EA-treated group were more intact compared to those in the model group. Additionally, synapses and the blood-brain barrier in the ischemic penumbra exhibited better preservation and greater mitochondrial abundance in the EA group (*n* = 4 per group) (Fig. 2, B; Supplementary Fig. 3, A).

Building on previous studies demonstrating that mitochondria from healthy primary astrocytes can be transferred to damaged neurons to enhance their survival and recovery [26], mitochondria were extracted from the ischemic penumbra of the cerebral cortex of EA-treated MCAO model rats. These mitochondria were stereotactically injected into the ischemic penumbra of MCAO model rats to evaluate mitochondrial transfer and its effects on neuronal apoptosis (*n* = 5 per group). Immunofluorescence staining showed that injected mitochondria aggregated near neurons in the ischemic penumbra (Fig. 2, C1). Statistical analysis revealed a significantly reduced neuronal apoptosis rate in the injection group compared to the control group (*P* < 0.01), indicating that active mitochondria after EA intervention exerted neuroprotective effects (Fig. 2, C2).

To further verify whether EA facilitates mitochondria transfer from astrocytes to damaged neurons in vivo, mitochondria in cultured astrocytes were labeled with MitoTracker and injected into the cerebral cortex of normal rats. Following this, MCAO modeling and EA treatment were performed to observe EA-mediated intercellular mitochondrial transfer (Fig. 2, D–E). A dose-response study was conducted by injecting varying volumes of astrocytes intracerebrally (*n* = 5 per group). The results showed a decreasing trend in neuronal apoptosis rates with increasing injection volumes (2 µL, 3 µL, and 5 µL), though differences were not statistically significant (Fig. 2, D1). The mitochondrial distribution area showed significant differences between the control group and all injection groups (*P* < 0.05), with the 5 µL group showing the largest distribution area albeit without significant differences compared to the 2 µL and 3 µL groups (Fig. 2, D2).

Based on these dose-response findings, 5 µL of astrocytes was uniformly injected into the rat brain, for subsequent experiments (*n* = 5 per group). Following MCAO modeling and EA treatment, the neuronal apoptosis rate in the EA group was significantly lower than in the MCAO group (*P* < 0.05) (Fig. 2, E1). While the mitochondrial distribution area in the EA group showed an increasing trend compared to the MCAO group, this difference was not statistically significant (Fig. 2, E2).

### In vitro study on the effect of EA pretreatment on mitochondrial transfer from astrocytes to neurons in MCAO rats

Previous research by Hayakawa et al. demonstrated that astrocytes can protect damaged neurons after ischemic stroke through intercellular mitochondrial transfer [11]. To replicate this and extend this process, we utilized a transwell co-culture system to assess mitochondrial transfer between neural cells under OGD conditions. This model allowed us to assess mitochondrial transfer between neural cells and its effects on neuronal apoptosis and cytotoxicity during OGD/R. Additionally, we conducted in vivo experiments simulating EA pretreatment to investigate whether this approach could enhance mitochondrial release from astrocytes.

To establish optimal OGD/R conditions, primary neurons extracted from neonatal rats were subjected to varying durations of OGD followed by 24 h of reoxygenation. Using CCK-8 viability assays and Annexin V-FITC staining, we identified OGD 2 h/R 24 h as the ideal condition. Under these parameters, neurons exhibited significant viability reduction compared to the control group but retained sufficient activity for effective intervention testing. MAP2 staining confirmed that 88.89% of cells were neurons (MAP2⁺/DAPI⁺), ensuring a high purity of neuronal cultures (Supplementary Fig. 4).

**Fig. 3:**
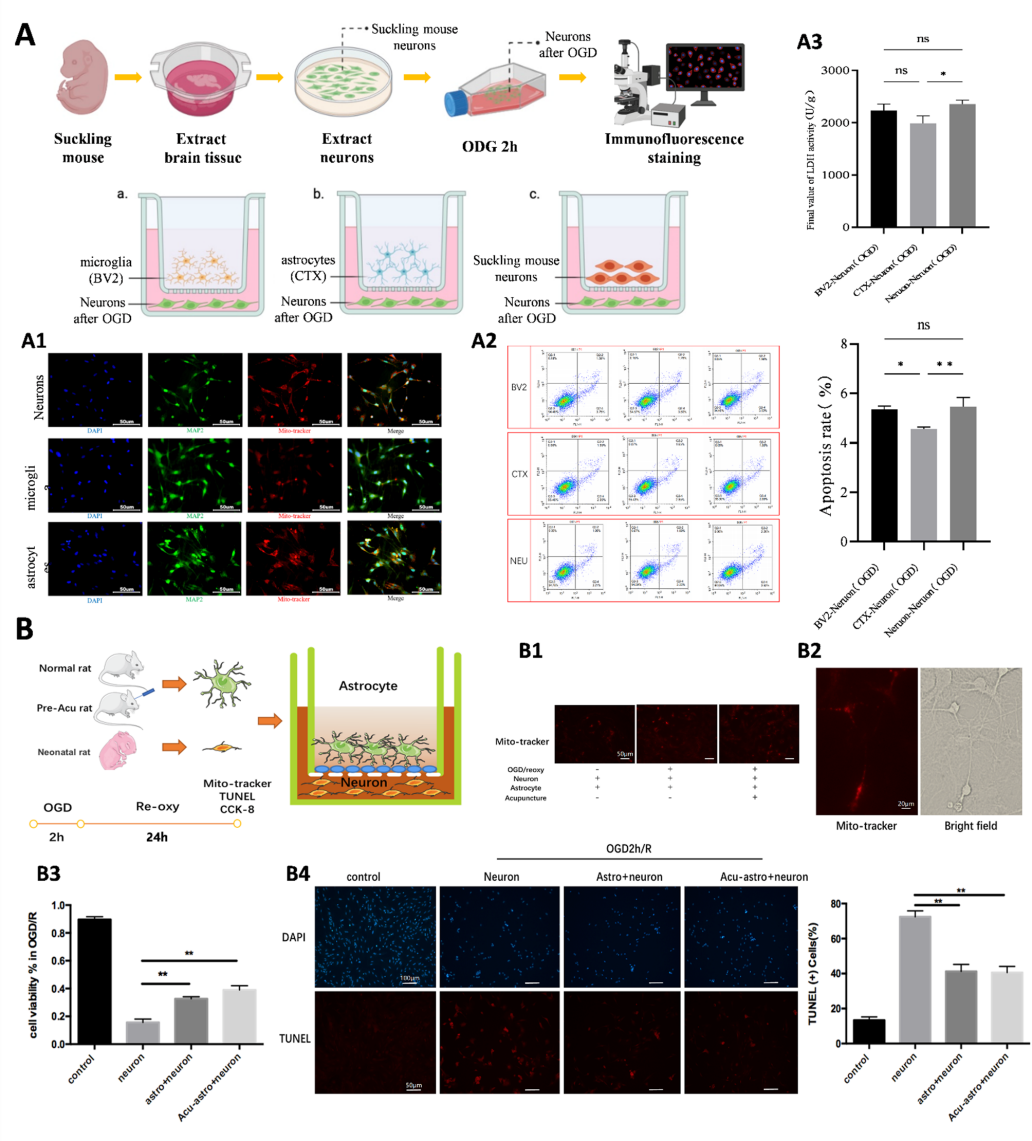
Study on the regulation of mitochondrial transcellular transfer by EA in MCAO rats. **(A)** In vitro experimental study on mitochondrial transcellular transfer in neural cells under OGD conditions. A1:Immunofluorescence staining of neurons, microglia and astrocytes in the lower chamber of Transwell. A2: Effect of (primary) neuronal group, microglia group and astrocyte group on the apoptosis rate of neuronal cells after OGD (flow cytometry) & Results of the apoptosis assay (Annexin V/PI double staining method). A3: Lactate dehydrogenase (LDH) cellular colorimetric assay results. **(B)** In vitro experimental study on EA preconditioning promoting mitochondrial release from astrocytes in the ischemic penumbra of the rat cerebral cortex (Acu-Astro + Neuron group, *n* = 6; Astro + Neuron group, *n* = 5; neuron group, *n* = 4).B1: Mito-tracker labeled astrocytes mitochondria transferred into neurons (Mito-tracker in red, Fluorescence microscopy at 20x magnification). B2: Mitochondrial fluorescence markers that appear in the axon of neurons (Fluorescence microscopy at 40x magnification). B3: Relative cell viability of neurons wre assessed by CCK-8 after astrocytes/Acu-astrocytes treatment after OGD/R injury (** neuron vs. astro + neuron, *P* < 0.01;**neuron vs. Acu-astrocytes, *P* < 0.01). B4: Effect of EA preconditioning on positive cells percentage of TUNEL staining in OGD/R (** neuron vs. astro + neuron, *P* < 0.01;**neuron vs. Acu-astrocytes, *P* < 0.01)

In the co-culture system, immunofluorescence staining revealed that neurons in the primary neuron and astrocyte groups contained more mitochondria than those in the microglia group, indicating active mitochondrial transfer from astrocytes to neurons during OGD/R (Fig. 3, A1). Annexin V/PI staining showed significantly lower neuronal apoptosis rates in the astrocyte group compared to the neuron and microglia groups (*P* < 0.05), suggesting a protective role of astrocytes. The LDH cytotoxicity assay further confirmed reduced neurotoxicity in the astrocyte group compared to the neuron group (*P* < 0.05), while no significant differences were observed between the microglia and neuron groups (Fig. 3, A2–A3). These findings indicate that astrocytes effectively transfer mitochondria to neurons during OGD/R, reducing neuronal apoptosis and cytotoxicity.

Next, we explored the effects of EA pretreatment on mitochondrial transfer. Astrocytes extracted from EA-pretreated rats were labeled with MitoTracker, and mitochondrial transfer was analyzed in the co-culture system under OGD/R conditions. Fluorescence microscopy revealed red MitoTracker signals within neuronal somas and axons in the EA-pretreated astrocyte group, suggesting successful mitochondrial transfer (Fig. 3, B1–B2).

Subsequent co-culture studies revealed higher neuronal survival rates in the EA-pretreated astrocyte group (Acu-Astro + Neuron group, *n* = 6) compared to the regular astrocyte group (Astro + Neuron group, *n* = 5), although the difference was not statistically significant. Neurons subjected to OGD/R alone exhibited the lowest survival rates, with significant differences compared to both astrocyte groups (*P* < 0.01) (Fig. 3, B3). TUNEL staining showed no significant differences in apoptosis rates between the Acu-Astro + Neuron and Astro + Neuron groups. However, neurons in the OGD/R-alone group (neuron group, *n* = 4) displayed significantly higher apoptosis rates compared to both astrocyte groups (*P* < 0.01) (Fig. 3, B4).

These findings suggest that astrocytes protect neurons during OGD/R by supplying energy via mitochondrial transfer. Furthermore, EA pretreatment enhances this protective mechanism, reducing neuronal apoptosis and increasing neuronal activity. This enhanced effect may be attributed to EA-induced promotion of the mitochondrial release from astrocytes, enabling more effective intracellular transfer.

### Transcriptomic and proteomic studies on the effects of EA pretreatment and EA intervention on the ischemic penumbra region in MCAO rats

To elucidate the molecular mechanisms underlying neuroprotection conferred by EA pretreatment and intervention proteomic and transcriptomic analyses were conducted to identify key proteins, signaling pathways, and targets in the ischemic penumbra of MCAO rats.

**Fig. 4:**
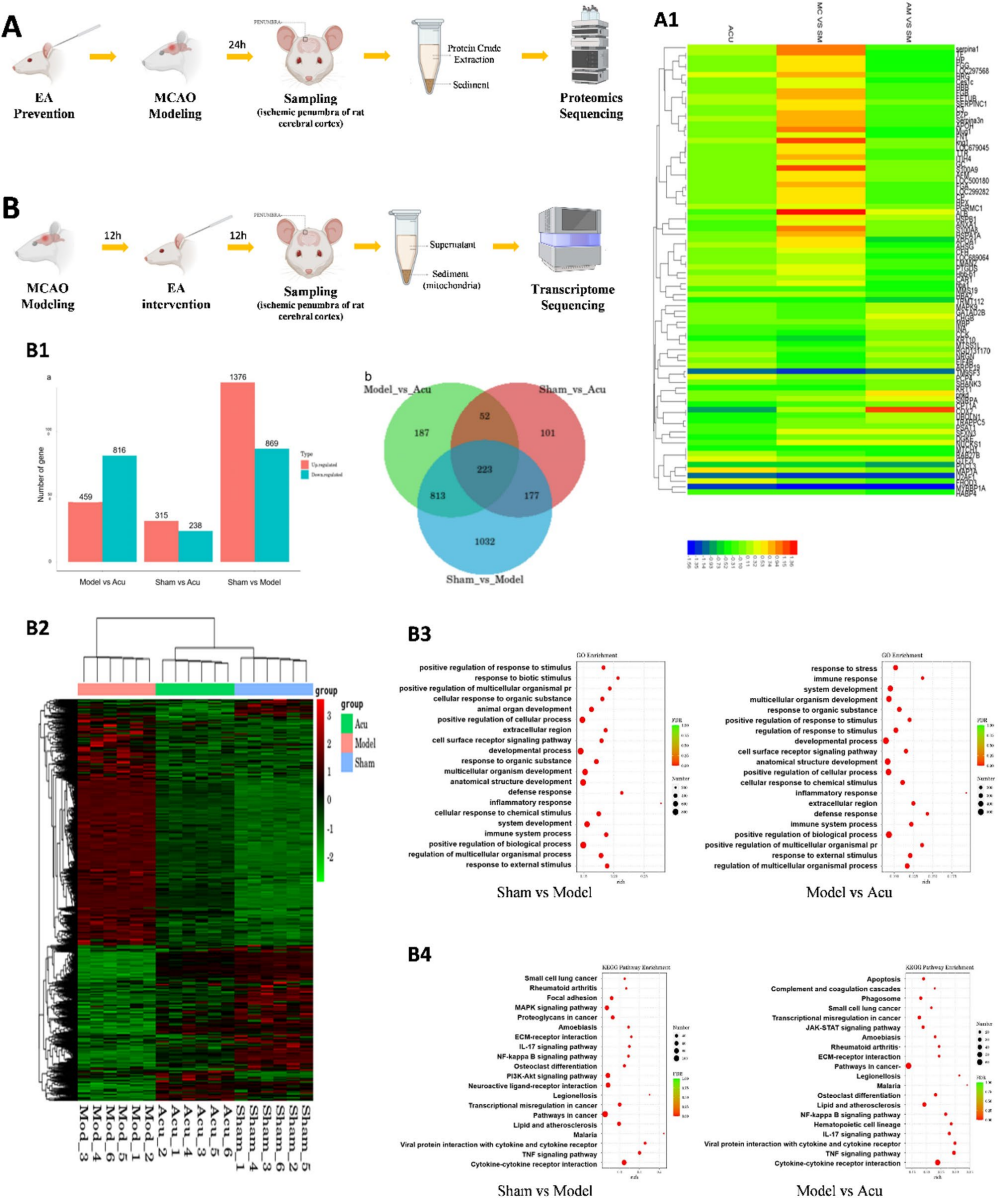
**(A)** Proteomic study on the effects of electroacupuncture preconditioning on the ischemic penumbra region of the brain cortex in MCAO rats (Sham group, *n* = 9; MCAO, *n* = 9; pre-acupuncture_MCAO group, *n* = 9; pre-acupuncture group, *n* = 9). A1: Cluster analysis of differentially expressed proteins (ACU: pre-acupuncture; SM: sham; MC: MCAO; AM: pre-acupuncture_MCAO). **(B)** Transcriptomic study on the effects of electroacupuncture intervention on the ischemic penumbra region of the brain cortex in MCAO rats (Sham group, *n* = 15; Model group, *n* = 15; Acu group, *n* = 15). B1: Statistical Analysis of Differential Expression Results (“**a”** shows the statistical results of differential expression analysis. The x-axis represents the comparison groups for the analysis, while the y-axis indicates the number of differentially expressed genes. Red represents upregulated genes, and green represents downregulated genes. “**b”** presents a Venn diagram of differentially expressed genes across groups, illustrating the intersections and unions of the Sham, Model, and Acu datasets. The overlapping regions of the circles indicate shared genes with similar expression changes, while the non-overlapping regions represent genes unique to each group). B2: Cluster analysis of differentially expressed genes. B3: GO enrichment analysis of differentially expressed genes. B4: KEGG enrichment analysis of differentially expressed genes

In the EA pretreatment experiment, proteins extracted from the cortical penumbra were analyzed for changes following ischemia-reperfusion injury and EA pretreatment (Sham group, *n* = 9; MCAO group, *n* = 9; pre-acupuncture_MCAO group, *n* = 9; pre-acupuncture group, *n* = 9). Using iTRAQ-based quantitative proteomics, a total of 5,656 proteins were identified. Screening criteria (fold change > 1.2 or < 0.83, and *P* < 0.05) revealed differentially expressed proteins across comparisons: MCAO vs. Sham (56 proteins, 28 upregulated, 28 downregulated), pre-acupuncture vs. Sham (54 proteins, 28 upregulated, 26 downregulated), pre-acupuncture_MCAO vs. MCAO (21 proteins, 9 upregulated, 12 downregulated), and pre-acupuncture_MCAO vs. Sham (46 proteins, 29 upregulated, 17 downregulated). Heatmap analysis revealed minimal changes in the pre-acupuncture group, while the MCAO group exhibited significant protein upregulation that normalized after EA pretreatment (Fig. 4, A1).

Key proteins, associated with the CD38 signaling pathway and calcium homeostasis—S100A9, Heat Shock Protein Beta-1 (HSPB1), and Annexin A1 (ANXA1)—showed notable normalization. S100A9, a calcium-binding protein involved in inflammation and calcium signaling, and CD38, critical for calcium transduction, were restored to baseline levels. HSPB1 supports calcium channel function under cellular stress, while ANXA1 facilitates calcium-dependent transmembrane transport. These findings suggest that EA pretreatment ameliorates ischemia-reperfusion injury in MCAO rats by promoting mitochondrial transfer through CD38-related calcium homeostasis.

In the EA intervention experiment, mitochondria isolated from the cortical penumbra were subjected to transcriptomic analysis via RNA-Seq. Differential gene expression was assessed using DESeq, with|log₂FoldChange| > 1 and *P* < 0.05 (Sham group, *n* = 15; Model group, *n* = 15; Acu group, *n* = 15). Compared to the Sham group, the model group exhibited 2,245 DEGs (1,376 upregulated, 869 downregulated genes), while the Acu group had 1,275 DEGs (459 upregulated, 816 downregulated). Between the Sham group and Acu groups there were 553 DEGs (315 upregulated, 238 downregulated) (Fig. 4, B1–B2).

Key DEGs related to EA-mediated mitochondrial effects included those governing cytoskeleton dynamics (Myo3b, Mfap5, Myh7, Tnnt2), intercellular communication (Gja5, Gjb6), GTPases (Gch1, Arhgap8), membrane integrity (Apoa2, Lcn2, Apold1, Apoc4), G protein-coupled receptors (Gpr6, Gng14, Rgs16, Rgs1), immune response (Tnfsf18, Il1b, Lilrb4, Tnfrsf14, Il2rb, Il6, Tlr1, Wnt6, Nfkbiz), apoptosis (Bcl2a1, AC135826.1, Ecscr), autophagy (Depp1), ATPases (Atp8b4, Atp2a3), chemokines (Ccl12, Ccl20, Ccl3), and ion regulation (S100a8, Kcng4, Calca, Rrad, Stc2).

Go analysis of EA’s interventional effects highlighted enrichment in intrinsic membrane components, transmembrane transporter activity, cell surface receptor signaling, immune response, and cytokine regulation pathways. KEGG pathway analysis demonstrated that EA significantly influenced apoptosis, phagosome formation, NF-κB signaling, IL-17 signaling, TNF signaling, cAMP signaling, and cytokine–cytokine receptor interaction pathways (Fig. 4, B3–B4).

### EA pretreatment promotes mitochondrial transfer in MCAO rats via the CD38-cADPR-Ca²⁺ pathway

CD38, a membrane-bound glycoprotein, regulates intracellular calcium dynamics through the synthesis and degradation of cyclic ADP-ribose (cADPR), a potent calcium activator. Previous studies, including those by Hayakawa et al., have highlighted the CD38-cADPR-Ca²⁺ signaling pathway as critical for glial mitochondrial release. Activation of this pathway increases extracellular mitochondrial production in astrocytes and facilitates mitochondrial transfer to neurons, enhancing neuronal survival and plasticity after stroke [11]. Our earlier findings indicated that EA pretreatment promotes mitochondrial release from astrocytes and improves neuronal survival. Proteomic analyses identified significant changes in key Ca²⁺-regulating proteins such as S100A9, HSPB1, and ANXA1, following EA pretreatment. These findings suggest that EA pretreatment modulates CD38-related pathways to promote mitochondrial transfer and alleviate ischemia-reperfusion injury in MCAO rats.

**Fig. 5:**
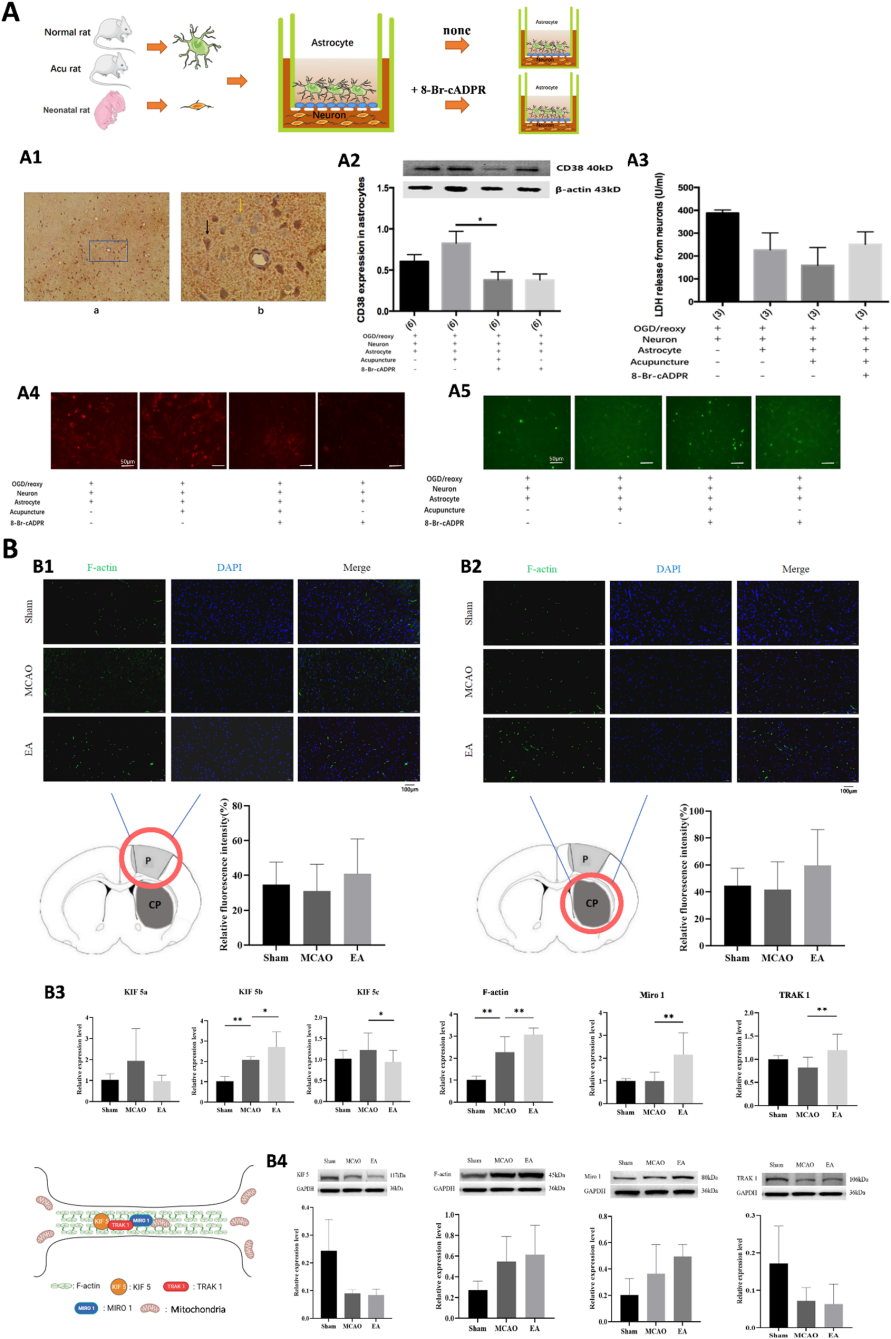
Mechanistic study on how EA promotes the release of mitochondria from astrocytes in the ischemic penumbra of the rat cerebral cortex. **A)** EA preconditioning promotes the release of mitochondria from astrocytes in the ischemic penumbra of the rat cerebral cortex by upregulating the expression of astrocytic CD38 and indirectly modulating the CD38-cADPR-Ca²⁺ pathway (*n* = 8 per group). A1: Immunohistochemical observation of the expression of CD38 on neurons and astrocytes. A2: The expression of CD38 in astrocyts with or without 8-Br-cADPR. Tested by Western blot (*n* = 4 per group, *P < 0.01). A3: The effect of 8-BR-CADPR on LDH release level of neurons after co-culture with EA-preconditioned astrocytes (*n* = 4 per group). A4: Antagonizing CD38 inhibits the release of mitochondria from EA-preconditioned astrocytes to neurons injured by OGD. A5: Effect of 8-Br-cADPR on intracellular Ca2 + in astrocyte after co-cultured in OGD condition. **(B)** Molecular mechanism study on the intercellular transfer of astrocytic mitochondria via the TNT pathway induced by EA in MCAO model rats. B1: Effect of EA on F-actin expression in P region (*n* = 5 per group). B2: Effect of EA on F-actin expression in CP region(*n* = 5 per group). B3: The effect of EA on the gene expression of F-actin, Miro 1, TRAK 1, KIF 5a, KIF5b and KIF5c (*n* = 4 per group, ^##^*P*<0.01 vs. Sham; *P<0.05, **P<0.01 vs. MCAO). B4: The effect of EA on the expression of F-actin, Miro 1, TRAK 1 and KIF 5 protein(*n* = 4 per group)

Immunohistochemistry in cortical penumbra tissue revealed varying levels of CD38 expression among cell types. Astrocytes exhibited higher CD38 expression (yellow arrows), followed by endothelial cells in small blood vessels, while neurons displayed lower expression (black arrows) (Fig. 5, A1). To further assess EA-induced effects on CD38-mediated mitochondrial transfer, astrocytes were extracted from EA-pretreated rats and co-cultured with neurons in a transwell model. Four experimental groups were established: neurons + astrocytes, neurons + astrocytes from EA-pretreated rats, neurons + EA-pretreated astrocytes with a CD38 antagonist (8-Br-cADPR), and neurons + astrocytes with a CD38 antagonist (*n* = 8 per group). All groups were subjected to 2 h of OGD followed by 24 h of reoxygenation. Western blot analysis revealed upregulated CD38 expression in astrocytes from the Neuron + Pre-treated Astrocyte group compared to the Neuron + Astrocyte group, while CD38 expression was significantly reduced in groups treated with the CD38 antagonist (*n* = 4 per group, *P* < 0.01) (Fig. 5, A2**).** To evaluate the neuroprotective effects of EA-pretreated astrocytes, lactate dehydrogenase (LDH) release was measured as marker of neuronal damage. LDH levels were lower in the Neuron + EA-pretreated Astrocyte group than in the Neuron + Astrocyte group, although not significantly different. LDH levels increased significantly in the presence of the CD38 antagonist, indicating reduced neuroprotection (*n* = 4 per group) (Fig. 5, A3).

To determine whether EA promotes mitochondrial transfer, astrocytic mitochondria were labeled with MitoTracker Red, and fluorescence microscopy was used to observed mitochondrial distribution in neuronal soma. The Neuron + Pre-treated astrocyte group exhibited significantly more mitochondrial fluorescence compared to the Neuron + Astrocyte group, while the addition of the CD38 antagonist significantly diminished mitochondrial transfer (Fig. 5, A4). These results strongly suggest that EA pretreatment promotes mitochondrial transfer from astrocytes to neurons via the CD38 signaling pathway.

To effects of EA pretreatment on Ca²⁺ activity in astrocytes were assessed using Fluo-3 AM. Fluorescence microscopy revealed increased intracellular Ca²⁺ activity in the Neuron + Pre-treated Astrocyte group compared to the Neuron + Astrocyte group. Although the CD38 antagonist reduced Ca²⁺ fluorescence intensity in the Neuron + EA-pretreated Astrocyte + CD38 antagonist group, levels remained higher than that in the Neuron + Astrocyte + CD38 antagonist group (Fig. 5, A5). This indicates that EA-enhanced Ca²⁺ activity in astrocytes is partially dependent on the CD38 pathway.

**Fig. 6:**
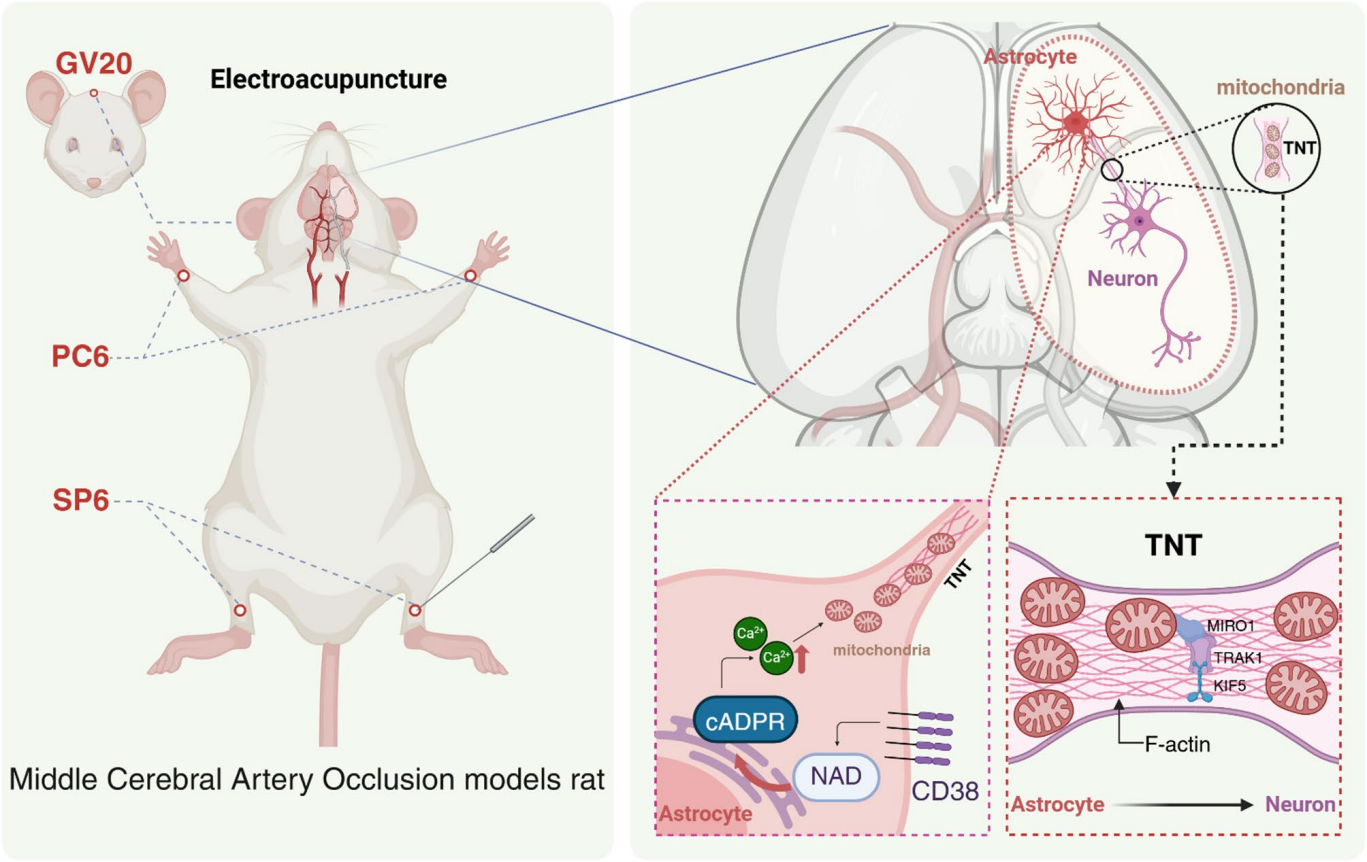
Mechanism diagram illustrating how electroacupuncture may promote the intercellular transfer of mitochondria from astrocytes in the brains of MCAO rats by regulating the CD38-cADPR-Ca²⁺ signaling pathway and the expression of TNT-related proteins (F-actin, Miro1, TRAK1, and KIF5b)

In summary, EA pretreatment promotes mitochondrial transfer from astrocytes to neurons by upregulating CD38 expression in astrocytes and enhancing intracellular Ca²⁺ activity. These effects are critical for neuronal survival and protection after ischemic injury. The partial reversal of these effects by the CD38 antagonist underscores the importance of the CD38-cADPR-Ca²⁺ pathway in the neuroprotection mechanisms mediated by EA pretreatment (Fig. 6).

### EA intervention promotes mitochondrial transfer in MCAO rats via upregulation of TNT pathway-related proteins

TNTs are specialized cellular structures rich in F-actin that form cytoplasmic bridges between cells, facilitating the transport of organelles, including mitochondria, from astrocytes to neurons [16]. TNT-mediated mitochondrial transfer relies on key regulatory proteins, such as Mitochondrial Rho GTPase 1 (Miro 1), Trafficking kinesin protein 1 (TRAK 1), and kinesin family member 5 (KIF5), which collectively enable mitochondrial movement along TNTs. Miro 1 anchors mitochondria to microtubules, while TRAK 1 links Miro 1 to KIF5, ensuring efficient intracellular transport [27–29].

Building on prior transcriptomic findings, we identified several genes in the MCAO model influenced by EA intervention, including Myo3b, Arhgap8, Gja5, Gjb6, Wnt6, S100a8, Ecscr, Calca, and Mfap5, all associated with TNT formation and function. These genes are involved in actin filament dynamics, cytoskeletal reorganization, and intercellular communication. To explore whether EA intervention promotes mitochondrial transfer via TNT regulation, we injected mitochondria-labeled astrocytes into the brains of rats subjected to MCAO, followed by EA treatment (*n* = 5 per group). Immunofluorescence staining revealed decreased F-actin fluorescence intensity in the P and CP regions of the MCAO group compared with the sham group. However, F-actin expression in the EA-treated group exhibited an upward trend relative to the MCAO group, though; however, the difference was not statistically significant (Fig. 5, B1-B2). These findings suggest a potential role for EA in enhancing F-actin expression.

RT-qPCR and Western blot analyses further assessed the expression of TNT-related proteins in the ischemic brains of MCAO rats. Results indicated significant upregulation of F-actin, Miro 1, TRAK 1, and KIF 5b in the EA group compared to the MCAO group (*P* < 0.05), suggesting EA facilitates mitochondrial through TNT pathways. Conversely, KIF 5a and KIF 5c exhibited either no significant changes or downward trends in response to EA, indicating their roles may be more complex or less directly involved in this mechanism (*n* = 4 per group) (Fig. 5, B3). Protein analysis corroborated these trends, showing increased F-actin and Miro 1 expression in both the MCAO and EA groups compared to the sham group, though differences did not reach statistical significance. TRAK 1 and KIF 5 expression trends mirrored this, displaying a decline in the MCAO and EA groups with no significant differences relative to controls (*n* = 4 per group) (Fig. 5, B4).

These findings suggest that EA intervention promotes mitochondrial transfer from astrocytes to neurons by upregulating critical TNT-associated proteins, including F-actin, Miro 1, TRAK 1, and KIF 5b. Despite the absence of statistically significant changes in some protein expression levels, the overall trends indicate that EA enhances TNT-mediated intercellular mitochondrial transfer, contributing to neuroprotection in ischemic brain injury. Further investigation is required to elucidate the precise molecular mechanisms governing this process (Fig. 6).

## Discussion

This study investigated the effects of EA preconditioning and intervention on cerebral ischemia-reperfusion injury in rats, emphasizing the regulatory role of EA in facilitating mitochondrial transfer from astrocytes to neurons. Using in vivo and in vitro experiments, we assessed the neuroprotective efficacy of EA by examining neurobehavioral scores, brain infarct volume, neuronal apoptosis, and mitochondrial function. By integrating findings with proteomic and transcriptomic analyses, this study explored the potential molecular mechanisms underlying EA’s neuroprotective effects.

In traditional Chinese medicine (TCM), “vital Qi” maintains health and resists external pathogens. is considered the fundamental force that maintains health and resists external pathogens. Modern scientific perspectives parallel this concept with mitochondrial function, which provides essential energy for cellular activities through ATP production. Studies have shown that EA exerts neuroprotective effects post-stroke by preserving mitochondrial structure, enhancing respiratory chain activity, reducing oxidative stress, and stabilizing membrane potential, as well as by promoting mitochondrial quality control and inhibiting apoptosis-related pathways [30]. These findings offer a modern explanation for the TCM view of acupuncture as a method to strengthen and protect Qi, though further research is needed to fully elucidate the mechanisms involved.

This study is the first to compare the effects of different frequencies of EA pretreatment (1, 3, and 5 sessions) on ischemia-reperfusion injury in rats. Results showed that 5 sessions of EA preconditioning yielded significant neuroprotective effects, including improved neurological functional scores, reduced infarct volume, and decreased neuronal apoptosis rates. Additionally, the timing of EA intervention was found to be critical, with the best outcomes observed when EA was administered 12 h post-MCAO. These findings align with previous research suggesting that intervention within 12–24 h after MCAO provides optimal benefits [31]. TEM analysis revealed that EA preserved neuronal and astrocytic morphology, reducing damage to cell membranes and organelles, consistent with prior reports of EA’s protective effects on neuronal integrity after ischemia-reperfusion injury [32, 33]. The structural integrity observed in our study suggests that EA has a protective effect on neuronal morphology after ischemia/reperfusion injury.

Astrocytes play a central role in supporting neurons, maintaining ionic homeostasis, and secreting cytokines such as NGF, IL-6, and TGF-β, which aid in neuronal repair and protection [34, 35]. Excessive astrocyte apoptosis, however, can negatively impact neurons. EA was found to enhance astrocytic function, reducing neuronal death and increasing activity under hypoxic and OGD conditions. These findings are consistent with prior studies indicating that EA stabilizes astrocyte morphology, inhibits apoptosis, and reduces neuroinflammation [36–38]. Furthermore, EA acts on the P2 × 7 receptors of astrocytes to alleviate pain responses [39], improve astrocyte structural abnormalities in a brain ischemia-induced depression model, and alleviate behavioral deficits [40]. These studies suggest that EA plays an important role in regulating astrocyte activity and morphology.

Mitochondrial dysfunction is a key contributor to ischemia-reperfusion injury, as increased intracellular Ca²⁺ leads to mitochondrial calcium overload, membrane potential disruption, and apoptosis [41]. Hayakawa et al. identified the CD38-cADPR-Ca²⁺ pathway as essential for astrocytic mitochondrial release, a finding supported by our study. EA preconditioning upregulated CD38 expression in astrocytes, enhanced intracellular Ca²⁺ activity, and promoted mitochondrial transfer to neurons. The application of a CD38 antagonist inhibited mitochondrial transfer and attenuated neuroprotection, further implicating this pathway in EA’s effects.

Mitochondrial transfer primarily through TNTs, which are enriched with F-actin and form cytoplasmic bridges between cells. Notably, Chen et al. [16] found that TNTs were the main pathway for material transfer from astrocytes to neurons. Transcriptomic analysis revealed that EA modulated genes related to TNT formation and function, including Myo3b, Arhgap8, Gja5, Gjb6, Wnt6, S100a8, Ecscr, Calca, and Mfap5. Furthermore, EA increased the expression of F-actin, Miro1, TRAK1, and KIF5b, key proteins involved in mitochondrial transport. While protein-level changes were not always significant, these findings suggest that EA enhances TNT-mediated mitochondrial transfer. Future studies should extend observation periods or employ sensitive detection methods to confirm these results.

This study has several limitations. Specific labeling of astrocytic mitochondria for in vivo observation remains a technical challenge. Future studies need to incorporate the latest research technologies to optimize the study design. Furthermore, some of the findings regarding EA’s effect of EA on mitochondrial transfer still require further exploration, such as the validation of other potential pathways through proteomics and transcriptomics, and the investigation of alternative pathways for mitochondrial entry into neurons. Additionally, discrepancies between gene and protein expression levels suggest that post-transcriptional regulation and degradation may influence the results. Expanding the scope of research to include other cell types, such as microglia, could provide a more comprehensive understanding of EA’s mechanisms in ischemic injury.

## Conclusion

This study demonstrated the neuroprotective effects of EA against brain ischemia-reperfusion injury, particularly through the facilitation of mitochondrial transfer from astrocytes to neurons, enhancement of mitochondrial quantity and function, and reduction of neuronal damage. The results suggest that EA exerts its neuroprotective effects by modulating the CD38-cADPR-Ca²⁺ signaling pathway and upregulating proteins associated with the TNT pathway, thereby enhancing intercellular mitochondrial transfer. These findings provide valuable insights into the mechanisms underlying EA’s neuroprotective role and highlight its potential as a therapeutic strategy for stroke. Future research should further elucidate the molecular mechanisms of EA-mediated mitochondrial transfer, validate protein expression changes, and investigate the contributions of other cell types to these processes.

## Electronic supplementary material

Below is the link to the electronic supplementary material.

Supplementary Material 1

## Data Availability

The datasets used and/or analysed during the current study are available from the corresponding author on reasonable request.

## References

[1] Global regional. National burden of stroke and its risk factors, 1990–2019: a systematic analysis for the global burden of disease study 2019. Lancet Neurol. 2021;20(10):795–820. 34487721 10.1016/S1474-4422(21)00252-0 PMC8443449

[2] Wu S, Wu B, Liu M, et al. Stroke in china: advances and challenges in epidemiology, prevention, and management. Lancet Neurol. 2019;18(4):394–405. 30878104 10.1016/S1474-4422(18)30500-3

[3] Jahan R, Saver J L, Schwamm L H, et al. Association between time to treatment with endovascular reperfusion therapy and outcomes in patients with acute ischemic stroke treated in clinical practice. Jama. 2019;322(3):252–63. 31310296 10.1001/jama.2019.8286 PMC6635908

[4] Catanese L, Tarsia J. Acute ischemic stroke therapy overview. Circ Res. 2017;120(3):541–58. 28154103 10.1161/CIRCRESAHA.116.309278

[5] He Z, Ning N, Zhou Q, et al. Mitochondria as a therapeutic target for ischemic stroke. Free Radic Biol Med. 2020;146:45–58. 31704373 10.1016/j.freeradbiomed.2019.11.005

[6] MontañO A, Staff I, Mccullough L D, et al. Community implementation of intravenous thrombolysis for acute ischemic stroke in the 3- to 4.5-hour window. Am J Emerg Med. 2013;31(12):1707–9. 24060324 10.1016/j.ajem.2013.08.032

[7] Sutherland B A, Minnerup J, Balami JS, et al. Neuroprotection for ischaemic stroke: translation from the bench to the bedside. Int J Stroke. 2012;7(5):407–18. 22394615 10.1111/j.1747-4949.2012.00770.x

[8] Iadecola C, Anrather J. Stroke research at a crossroad: asking the brain for directions. Nat Neurosci. 2011;14(11):1363–8. 22030546 10.1038/nn.2953 PMC3633153

[9] Dawson T M Dawsonvl. Mitochondrial mechanisms of neuronal cell death: potential therapeutics. Annu Rev Pharmacol Toxicol. 2017;57:437–54. 28061689 10.1146/annurev-pharmtox-010716-105001 PMC11323062

[10] Davis C H, KIM K Y, Bushong E A, et al. Transcellular degradation of axonal mitochondria. Proc Natl Acad Sci U S A. 2014;111(26):9633–8. 24979790 10.1073/pnas.1404651111 PMC4084443

[11] Hayakawa K, Esposito E, Wang X, et al. Transfer of mitochondria from astrocytes to neurons after stroke. Nature. 2016;535(7613):551–5. 27466127 10.1038/nature18928 PMC4968589

[12] Alarcon-Martinez L, Villafranca-Baughman D, Quintero H, et al. Interpericyte tunnelling nanotubes regulate neurovascular coupling. Nature. 2020;585(7823):91–5. 32788726 10.1038/s41586-020-2589-x

[13] Berridge M V, Schneider R T, Mcconnell MJ. Mitochondrial transfer from astrocytes to neurons following ischemic insult: guilt by association??. Cell Metab. 2016;24(3):376–8. 27626198 10.1016/j.cmet.2016.08.023

[14] Wu B B, Leung K T, Poon E N. Mitochondrial-Targeted therapy for Doxorubicin-Induced cardiotoxicity. Int J Mol Sci, 2022, 23(3). 10.3390/ijms23031912 PMC8837080 35163838

[15] Han X. Wang X. Opportunities and challenges in tunneling nanotubes research: how Far from clinical application??. Int J Mol Sci, 2021, 22(5). 10.3390/ijms22052306 PMC7956326 33669068

[16] Chen J, Cao J. Astrocyte-to-neuron transportation of enhanced green fluorescent protein in cerebral cortex requires F-actin dependent tunneling nanotubes. Sci Rep. 2021;11(1):16798. 34408233 10.1038/s41598-021-96332-5 PMC8373867

[17] Choi S H. Who traditional medicine strategy and activities. Standardization with evidence-based approaches. J Acupunct Meridian Stud. 2008;1(2):153–4. 20633469 10.1016/S2005-2901(09)60037-6

[18] Chen F, Qi Z, Luo Y, et al. Non-pharmaceutical therapies for stroke: mechanisms and clinical implications. Prog Neurobiol. 2014;115:246–69. 24407111 10.1016/j.pneurobio.2013.12.007 PMC3969942

[19] Zhu W, Jia Q, Ferreira A C, et al. Acupuncture for ischemic stroke: where are we now?. Acupunct Herb Med. 2024;4(1):36–55.

[20] Wang Q, Wang F, Li X, et al. Electroacupuncture pretreatment attenuates cerebral ischemic injury through α7 nicotinic acetylcholine receptor-mediated Inhibition of high-mobility group box 1 release in rats. J Neuroinflammation. 2012;9:24. 22277256 10.1186/1742-2094-9-24 PMC3297509

[21] Xiong L Z, Yang J, Wang Q, et al. Involvement of delta-and mu-opioid receptors in the delayed cerebral ischemic tolerance induced by repeated electroacupuncture preconditioning in rats. Chin Med J (Engl). 2007;120(5):394–9. 17376310

[22] Sun Y, Li J, Georgi R, et al. Effects of acupuncture on angiogenesis-associated factor expression in ischemic brain tissue following cerebral infarction in rats. Acupunct Herb Med. 2023;3(1):46–54.

[23] Xing Y, Zhang M, Li W B, et al. Mechanisms involved in the neuroprotection of electroacupuncture therapy for ischemic stroke. Front Neurosci. 2018;12:929. 30618558 10.3389/fnins.2018.00929 PMC6297779

[24] Li B, Tian J P, Zhang S, et al. [Effect of bloodletting acupuncture at twelve jing-well points of hand on microcirculatory disturbance in mice with traumatic brain injury]. Zhongguo Zhen Jiu. 2019;39(10):1075–80. 31621260 10.13703/j.0255-2930.2019.10.012

[25] Shahjouei S, Cai P Y, Ansari S, et al. Middle cerebral artery occlusion model of stroke in rodents: A Step-by-Step approach. J Vasc Interv Neurol. 2016;8(5):1–8. 26958146 PMC4762402

[26] Lee E H, Kim M, Ko Sh, et al. Primary astrocytic mitochondrial transplantation ameliorates ischemic stroke. BMB Rep. 2023;56(2):90–5. 36195567 10.5483/BMBRep.2022-0108 PMC9978364

[27] Ahmad T, Mukherjee S, Pattnaik B, et al. Miro1 regulates intercellular mitochondrial transport & enhances mesenchymal stem cell rescue efficacy. Embo J. 2014;33(9):994–1010. 24431222 10.1002/embj.201386030 PMC4193933

[28] Brickley K, Stephenson F A. Trafficking Kinesin protein (TRAK)-mediated transport of mitochondria in axons of hippocampal neurons. J Biol Chem. 2011;286(20):18079–92. 21454691 10.1074/jbc.M111.236018 PMC3093881

[29] Chang K T, Niescier R F, Min KT. Mitochondrial matrix Ca2 + as an intrinsic signal regulating mitochondrial motility in axons. Proc Natl Acad Sci U S A. 2011;108(37):15456–61. 21876166 10.1073/pnas.1106862108 PMC3174631

[30] Li Y X, Zhang R C, Cheng A X, et al. Advances of research on mechanisms of acupuncture underlying improvement of ischemic stroke by regulating neuronal mitochondria. Zhen Ci Yan Jiu. 2024;49(1):71–8. 38239141 10.13702/j.1000-0607.20220909

[31] Li Z, Yang M, Lin Y, et al. Electroacupuncture promotes motor function and functional connectivity in rats with ischemic stroke: an animal resting-state functional magnetic resonance imaging study. Acupunct Med. 2021;39(2):146–55. 32576025 10.1177/0964528420920297

[32] Liu C L, Siesjö BK. Hu B R. Pathogenesis of hippocampal neuronal death after hypoxia-ischemia changes during brain development. Neuroscience. 2004;127(1):113–23. 15219674 10.1016/j.neuroscience.2004.03.062 PMC3518049

[33] Fan Y, Wang R, Zhang C, et al. [Effect of high frequency electrotherapy on caspase-3 and ultra microstructure of hippocampus in rats following cerebral ischemia/reperfusion]. Zhong Nan Da Xue Xue Bao Yi Xue Ban. 2017;42(1):21–5. 28216493 10.11817/j.issn.1672-7347.2017.01.004

[34] Verkhratsky A. Physiology of astroglia. Physiol Rev. 2018;98(1):239–389. 29351512 10.1152/physrev.00042.2016 PMC6050349

[35] Liu Z. Astrocytes, therapeutic targets for neuroprotection and neurorestoration in ischemic stroke. Prog Neurobiol. 2016;144:103–20. 26455456 10.1016/j.pneurobio.2015.09.008 PMC4826643

[36] Allaman I, Bélanger M, Magistretti PJ. Astrocyte-neuron metabolic relationships: for better and for worse. Trends Neurosci. 2011;34(2):76–87. 21236501 10.1016/j.tins.2010.12.001

[37] Lin SS, Chen B Zhoub, J, et al. Electroacupuncture prevents astrocyte atrophy to alleviate depression. Cell Death Dis. 2023;14(5):343. 37248211 10.1038/s41419-023-05839-4 PMC10227075

[38] Zhao H, Zong X, Li L, et al. Electroacupuncture inhibits neuroinflammation induced by astrocytic necroptosis through RIP1/MLKL/TLR4 pathway in a mouse model of spinal cord injury. Mol Neurobiol. 2024;61(6):3258–71. 37982922 10.1007/s12035-023-03650-y

[39] Zhao W, Liu S L, Lin SS, et al. Astrocytic P2X7 receptor in retrosplenial cortex drives electroacupuncture analgesia. Purinergic Signal; 2024. 10.1007/s11302-024-10043-w PMC12454730 39222236

[40] Wang J, Deng X, Jiang J, et al. Evaluation of electroacupuncture as a non-pharmacological therapy for astrocytic structural aberrations and behavioral deficits in a post-ischemic depression model in mice. Front Behav Neurosci. 2023;17:1239024. 37700911 10.3389/fnbeh.2023.1239024 PMC10493307

[41] Halestrap A P, Richardson A P. The mitochondrial permeability transition: a current perspective on its identity and role in ischaemia/reperfusion injury. J Mol Cell Cardiol. 2015;78:129–41. 25179911 10.1016/j.yjmcc.2014.08.018

