# Supplementary material for: Mechanisms of electroacupuncture-induced neuroprotection in acute stroke rats: the role of astrocyte-mediated mitochondrial transfer
Source: Cell Commun Signal. 2025 Jul 1;23:316. doi: 10.1186/s12964-025-02287-9 (PMC12219609; doi:10.1186/s12964-025-02287-9)
Supplement: Supplementary file 1 — Supplementary Material 1 [file 12964_2025_2287_MOESM1_ESM.docx]

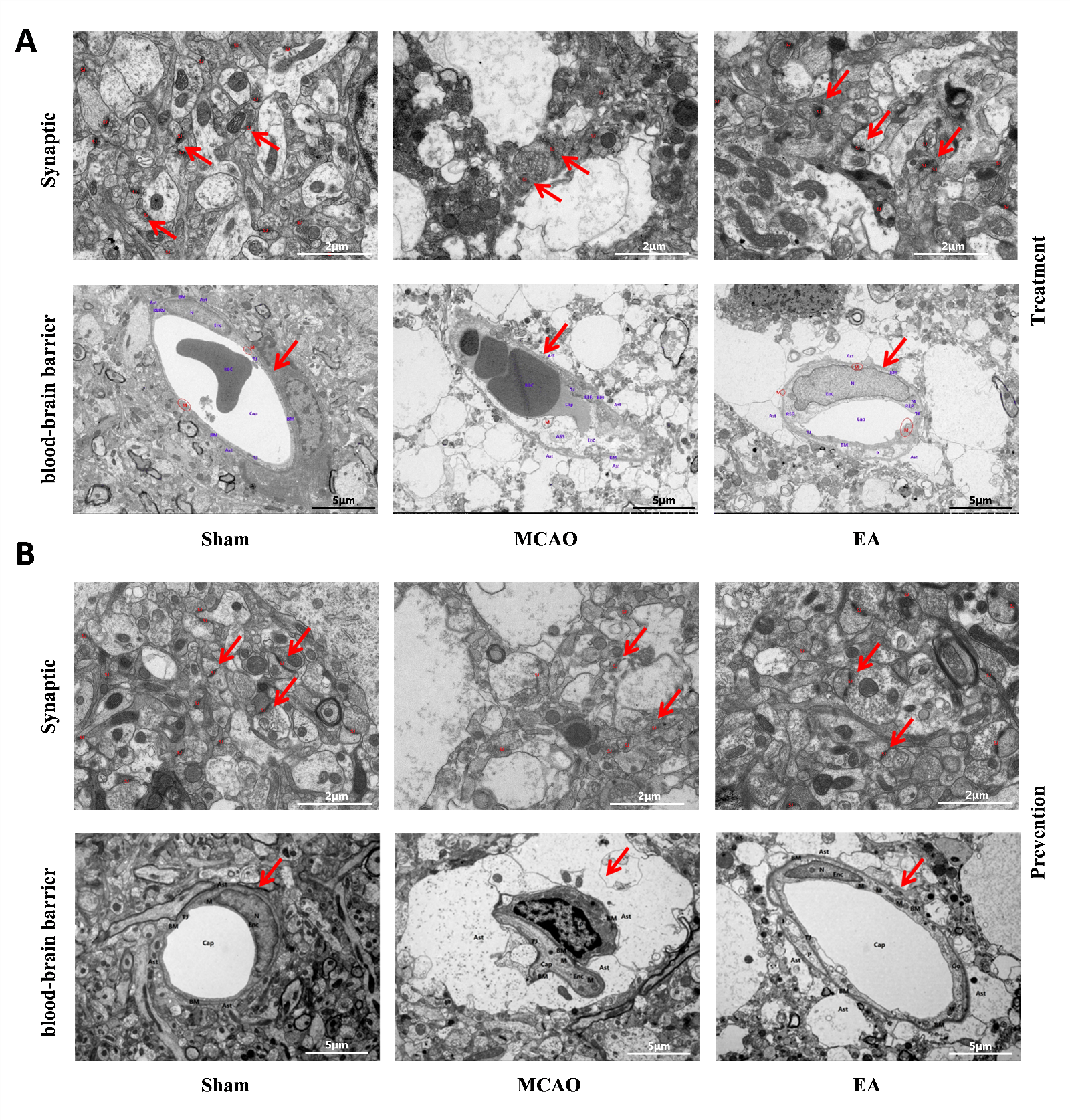


**Supplementary Fig. 1:** Electron micrographs showing the effects of EA pretreatment and EA intervention on the morphology and structure of synaptic and blood-brain barrier in the ischemic penumbra of the cerebral cortex in MCAO model rats.


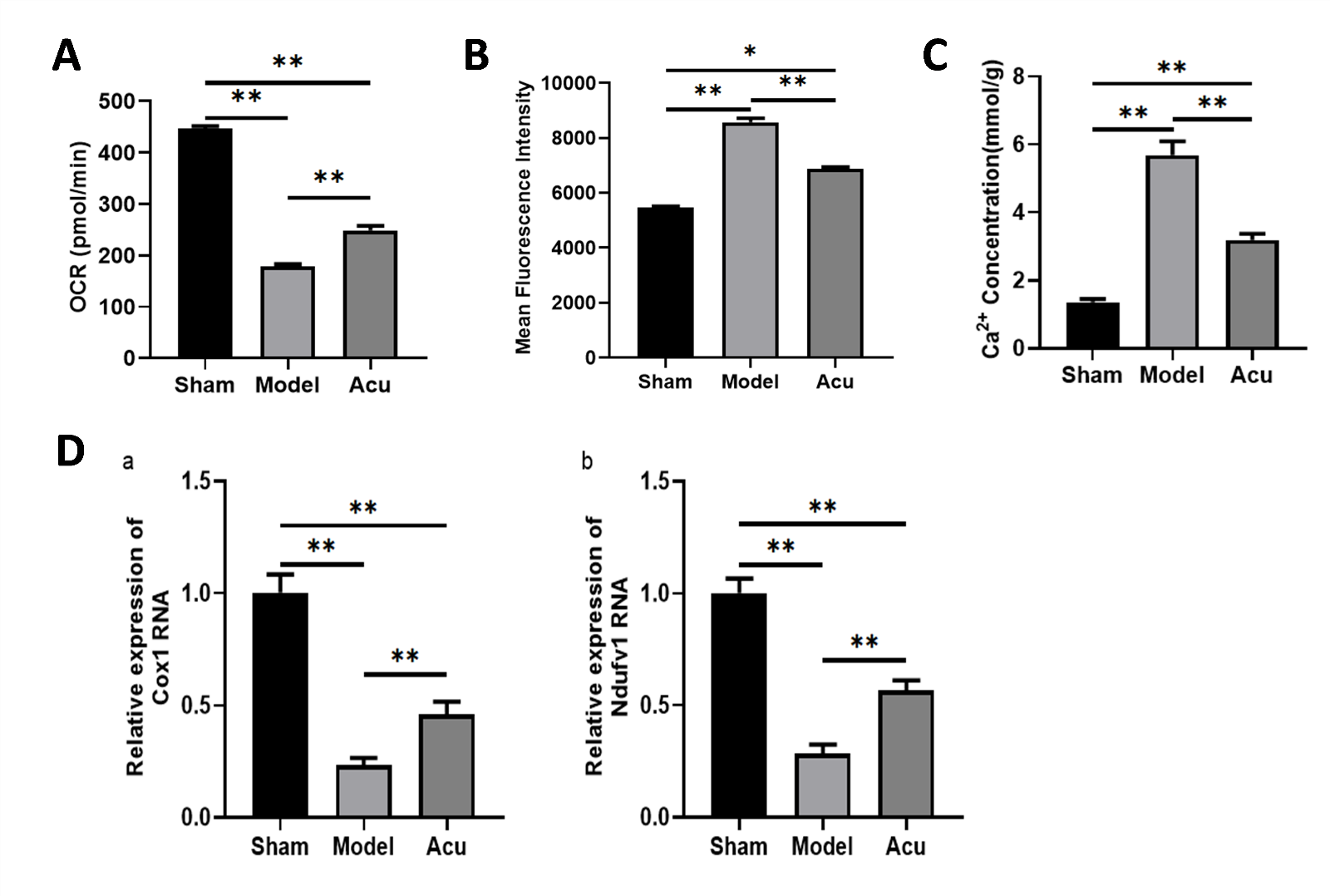


**Supplementary Fig. 2:** The effects of electroacupuncture on mitochondrial respiration rate (A), mitochondrial ROS fluorescence intensity (B), calcium ion concentration (C), and mitochondrial mtDNA (D) in the cortical ischemic penumbra of MCAO model rats.


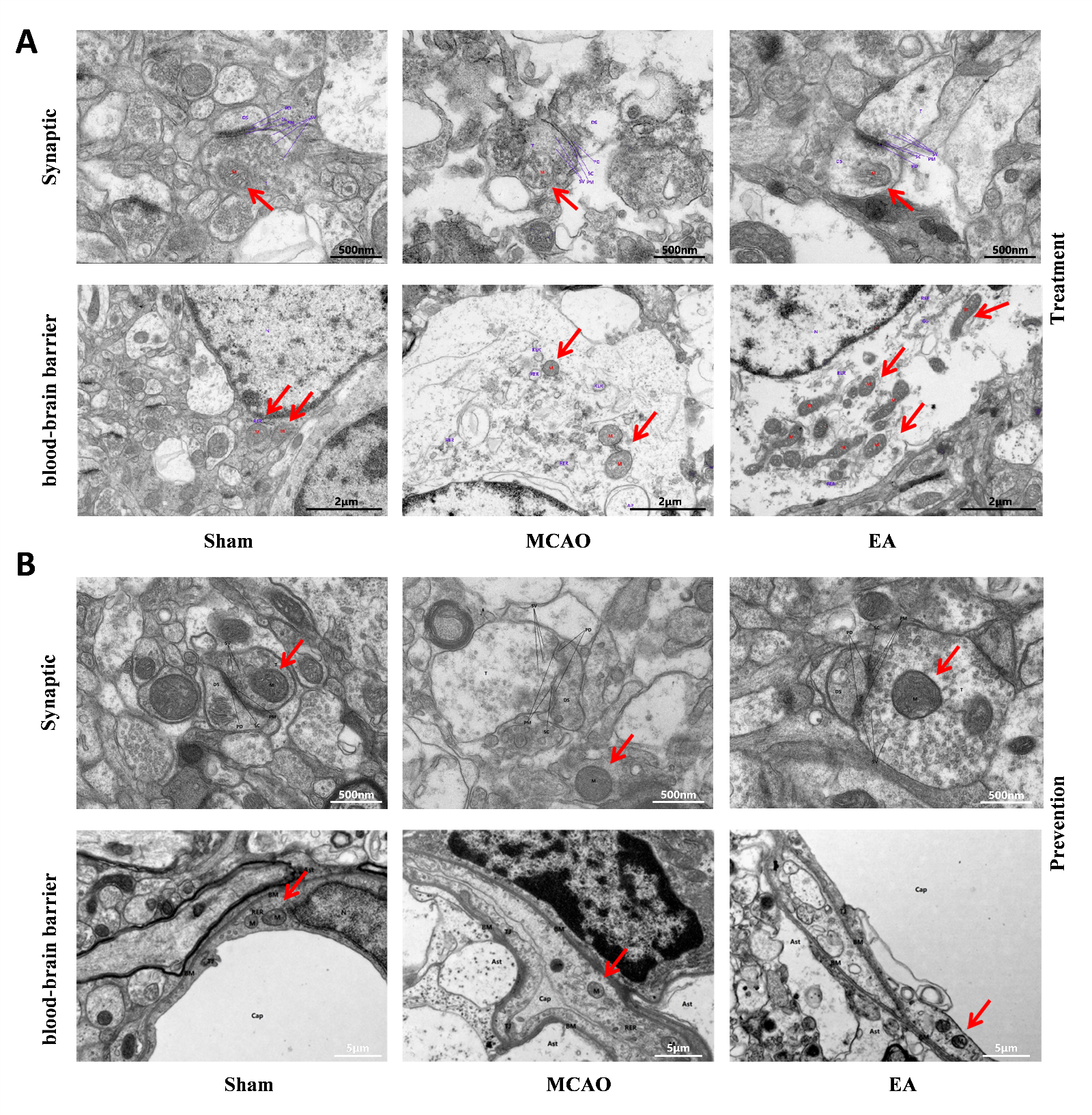


**Supplementary Fig. 3:** Electron micrographs showing the effects of EA intervention and EA preconditioning on the morphological structure of mitochondria near synapses and the blood-brain barrier in the ischemic penumbra of the cerebral cortex in MCAO model rats.


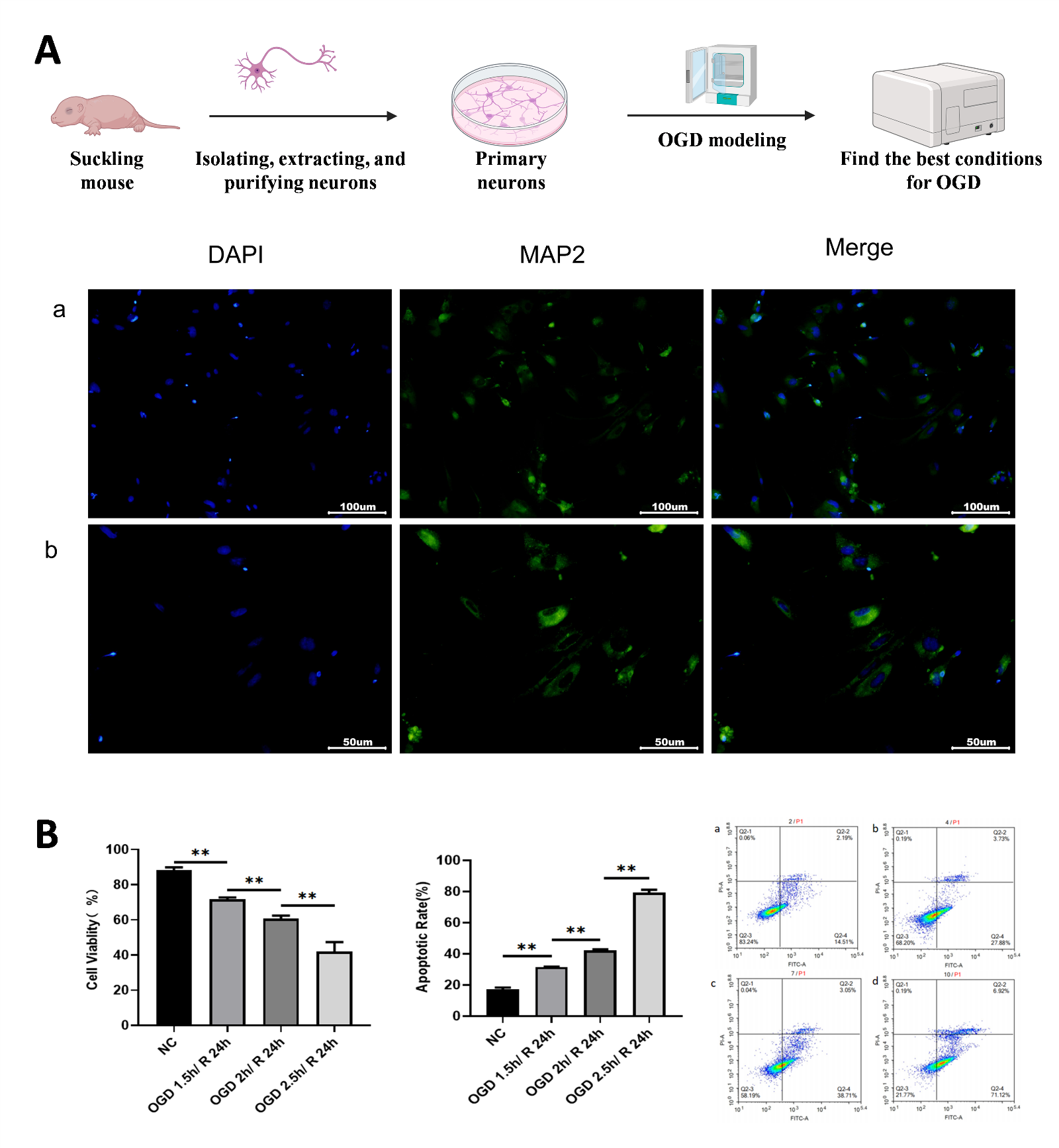


**Supplementary Fig. 4:** The effects of different durations of oxygen-glucose deprivation/reoxygenation (OGD/R) conditions on primary neurons.


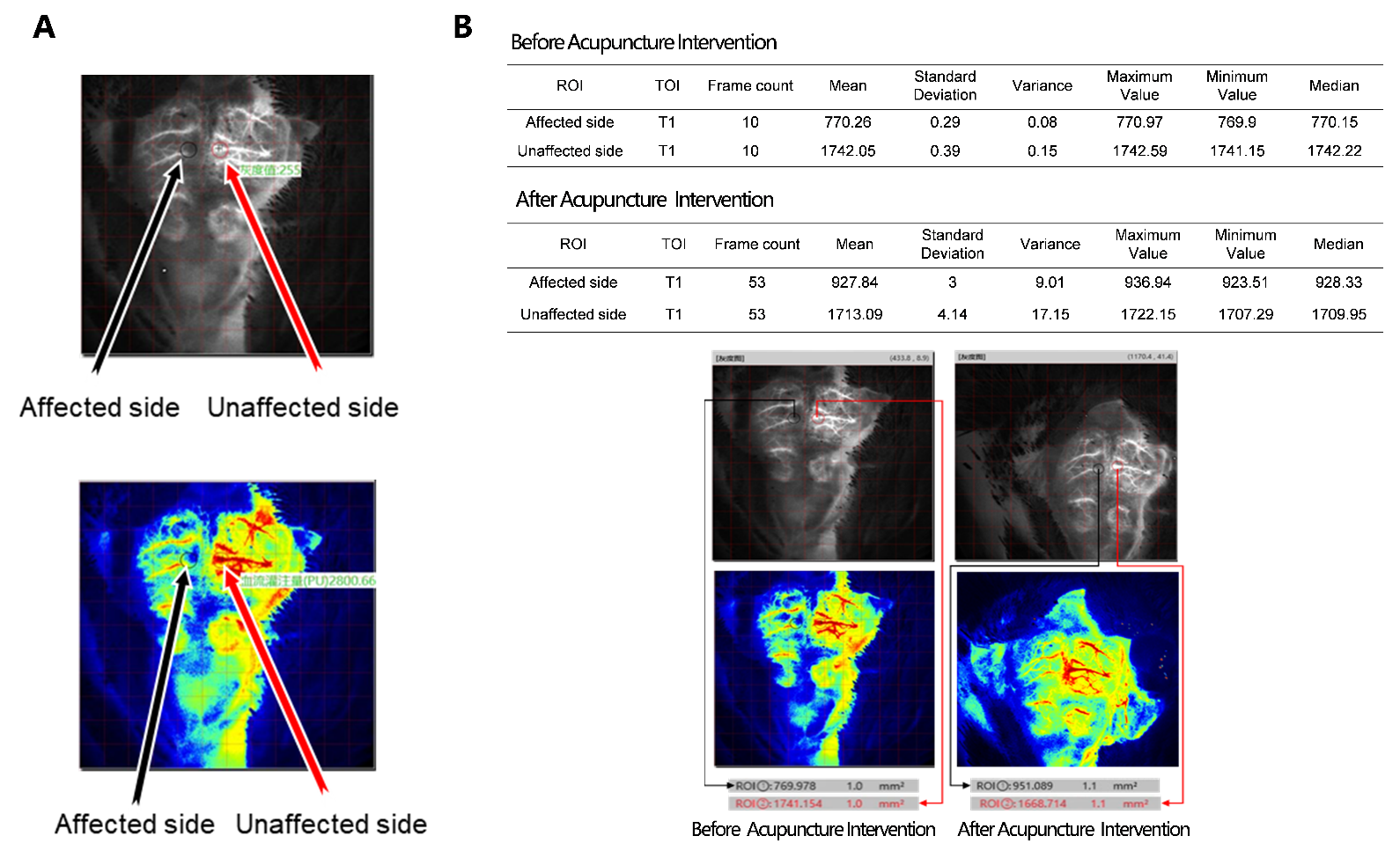


**Supplementary Fig. 5:** A: Blood Flow Changes in the Bilateral Middle Cerebral Arteries of Rats Post-Modeling. B: Comparison and Changes in Blood Flow of Bilateral Middle Cerebral Arteries in Rats Before and After Acupuncture Intervention
